# A mosquito juvenile hormone binding protein (mJHBP) regulates the activation of innate immune defenses and hemocyte development

**DOI:** 10.1371/journal.ppat.1008288

**Published:** 2020-01-21

**Authors:** Il Hwan Kim, Julio César Castillo, Azadeh Aryan, Inés Martin-Martin, Marcela Nouzova, Fernando G. Noriega, Ana Beatriz F. Barletta, Eric Calvo, Zachary N. Adelman, José M. C. Ribeiro, John F. Andersen

**Affiliations:** 1 NIH/NIAID Laboratory of Malaria and Vector Research, 12735 Twinbrook Parkway, Rockville, MD, United States of America; 2 Department of Entomology and AgriLife Research, Texas A&M University, College Station, United States of America; 3 Department of Biological Sciences and Biomolecular Science Institute, Florida International University, Miami, United States of America; 4 Institute of Parasitology, Biology Centre CAS, Ceske Budejovice, Czech Republic; University of Georgia, UNITED STATES

## Abstract

Insects rely on the innate immune system for defense against pathogens, some aspects of which are under hormonal control. Here we provide direct experimental evidence showing that the juvenile hormone-binding protein (mJHBP) of *Aedes aegypti* is required for the regulation of innate immune responses and the development of mosquito blood cells (hemocytes). Using an mJHBP-deficient mosquito line generated by means of CRISPR-Cas9 gene editing technology we uncovered a mutant phenotype characterized by immunosuppression at the humoral and cellular levels, which profoundly affected susceptibility to bacterial infection. Bacteria-challenged mosquitoes exhibited significantly higher levels of septicemia and mortality relative to the *wild type* (*WT*) strain, delayed expression of antimicrobial peptides (AMPs), severe developmental dysregulation of embryonic and larval hemocytes (reduction in the total number of hemocytes) and increased differentiation of the granulocyte lineage. Interestingly, injection of recombinant wild type mJHBP protein into adult females three-days before infection was sufficient to restore normal immune function. Similarly, injection of mJHBP into fourth-instar larvae fully restored normal larval/pupal hemocyte populations in emerging adults. More importantly, the recovery of normal immuno-activation and hemocyte development requires the capability of mJHBP to bind JH III. These results strongly suggest that JH III functions in mosquito immunity and hemocyte development in a manner that is perhaps independent of canonical JH signaling, given the lack of developmental and reproductive abnormalities. Because of the prominent role of hemocytes as regulators of mosquito immunity, this novel discovery may have broader implications for the understanding of vector endocrinology, hemocyte development, vector competence and disease transmission.

## Author summary

There are many unanswered questions concerning the nature of immune responses of mosquitoes to bacteria, viruses and parasites. This is important because a variety of human pathogens are transmitted by mosquitoes during the process of consuming blood. Much of mosquito physiology is under the control of hormones and we aim to understand a potential role for an important hormone known as juvenile hormone in anti-bacterial immunity. We have produced a strain of the mosquito, *Aedes aegypti*, that is deficient in the production of a protein that circulates in the blood while carrying juvenile hormone. This strain is shown to have less ability to control bacterial infection, to have lower levels of proteins involved in immunity and to have smaller numbers of blood cells that are known to be important in the mosquito immune response. If the protein is administered to the deficient strain by injection, the immune response and blood cell numbers return to near-normal levels. Other results suggest that the association of the protein with juvenile hormone is important for its ability to function in the immune system. Overall, this study describes an important new protein regulator of mosquito immunity and a potential role of juvenile hormone in this process.

## Introduction

The insect immune system is activated through a variety of pattern recognition receptors that trigger the Toll, IMD and JAK/STAT signaling pathways leading to the production of effector molecules. These molecules include antimicrobial peptides (AMPs), complement-like proteins, nitric oxide, and components of the pro-phenoloxidase cascade [1]. The ability to respond to microbial signals is known to be hormonally regulated. In *Drosophila melanogaster*, 20-hydroxyecdysone (20E) controls the expression of *PGRP-LC* which encodes a key pattern recognition receptor of the immune deficiency (IMD) pathway leading to the production of the AMPs cecropin A1, attacin A and defensin A [2]. This hormone also regulates expression of the AMPs diptericin, drosomycin and metchnikowin in a non-PGRP-LC-dependent manner [3]. It has also been established that cellular immunity is under hormonal control. *D*. *melanogaster* hemocytes require 20E for the acquisition of normal chemotactic and phagocytic behavior [4]. LRIM9, a pattern recognition protein involved in antimalarial activity is upregulated by injected 20E in *An*. *gambiae* [5] and recently, physiological systems regulated by 20E have been implicated in the growth and development of malaria parasites in the mosquito [6]. Additionally, the pro-phenoloxidase locus of *Anopheles* mosquitoes has been shown to be under the control of 20E [7]. The involvement of juvenile hormone (JH) in insect immunity has been less intensively investigated than 20E, but one study in *D*. *melanogaster* has shown that JH and JH analogs negatively regulate the production of AMPs after bacterial infection in Schneider S2* cells and *in vivo* [8].

Recently, a circulating JH binding protein, mJHBP, was identified in *Aedes aegypti* that differs structurally from the well characterized form found in lepidopteran larvae [9]. The protein is related to the salivary D7 proteins [10] and consists of two modified odorant-binding protein domains with a ligand binding site in the N-terminal domain that can stereoselectively accommodate a single molecule of JH III [9]. It is found only in pupae and adults, ruling out a role in JH regulation of larval development. JH is known to have essential functions in the adult female where it promotes maturation of the ovary prior to blood feeding and plays a regulatory role in linking the nutritional state of the female with blood meal-dependent reproductive development [11]. Titers of the hormone have been measured and indicate a peak of synthesis early in adulthood corresponding to the previtellogenic development of the ovary [12]. Here, we have examined the function of mJHBP in an mJHBP-deficient mutant generated using CRISPR-Cas9-mediated gene knockout technology. Rather than showing an effect on nutrition or ovarian development, we demonstrate that the mutant is defective in its antibacterial response and shows dramatically altered hemocyte numbers and hemocyte population structure, suggesting that JH may be regulating mosquito immunity and hematopoiesis through an unconventional mechanism.

## Results

### *Aedes aegypti* mJHBP is expressed only in pupal and adult stage mosquitoes

**Fig 1:**
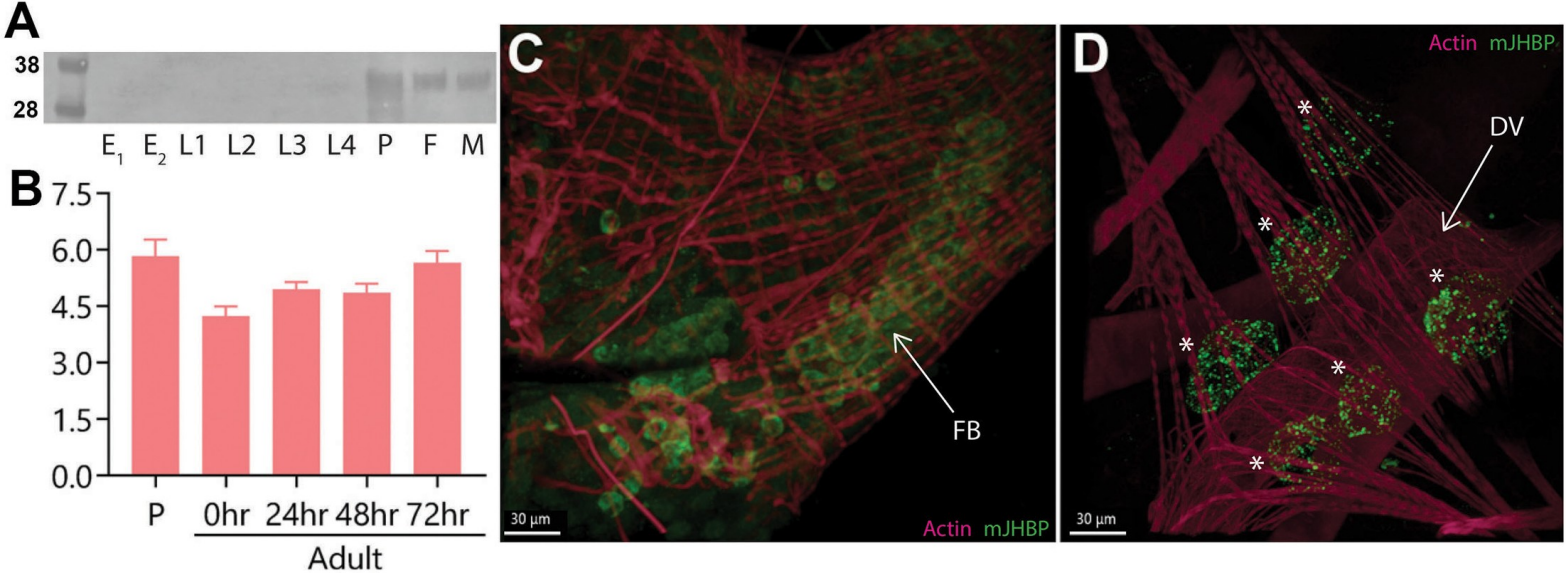
Expression, quantification and detection of mJHBP in *Ae*. *aegypti*. **A.** Detection of mJHBP protein in developmental stages by SDS-PAGE (NuPage) and Western blotting of whole mosquito homogenates (protein standard sizes in kilodaltons are shown on left). E1: Embryo (n = 20), E2: Egg (n = 20), L1: 1^st^ instar larvae (n = 20), L2: 2^nd^ instar larvae (n = 20), L3: 3^rd^ instar larvae (n = 20), L4: 4^th^ instar larvae (n = 20), P: pupae (n = 5), F: adult female (n = 5), M: adult male (n = 5). Samples were reduced with NuPAGE sample reducing agent (stabilized dithiothreitol) prior to electrophoresis. Molecular size standards are shown to the left of the gel. **B.** Quantitation of *Ae*. *aegypti* mJHBP protein in individual pupae (P) and adult female mosquitoes (0, 24, 48 and 72 h post emergence) measured by ELISA. Each bar represents the mean (ng / individual ± SEM) quantity of mJHBP from 15 mosquitoes (3 replicates of 5 insects in each experiment). There are no statistical differences between timepoints or developmental stages. **C.** Visualization of *Ae*. *aegypti* mJHBP protein in the fat body (FB) by confocal microscopy. mJHBP, green; actin, magenta. **D.** Visualization of *Ae*. *aegypti* mJHBP protein in pericardial cells (asterisks, DV = dorsal vessel) by confocal microscopy. mJHBP, green; actin, magenta; Scale bar, 30 μm.

mJHBP is present in the cell-free fraction of hemolymph from male and female adult *Ae*. *aegypti* [9]. The protein has also been detected in the pupal stage, but not in the late larval stages [9]. To obtain a more detailed picture of the developmental expression of mJHBP, homogenates of embryos, eggs, larvae, pupae and adult females were examined by immunoblotting, immunofluorescence microscopy, or both (Figs 1, S1B, S1C and S2A). mJHBP was also quantified in these homogenates using ELISA methods. On immunoblots, mJHBP was detected only in the pupal and adult stages of *Ae*. *aegypti*, agreeing with previous results [9] (Figs 1A and S1). Larvae, eggs and embryos did not contain observable levels of the protein (Figs 1A and S2). Quantification by ELISA showed approximately 5.8 ng of mJHBP per individual in late pupae, and 4.2–5.6 ng in adults 0 to 72 h post adult emergence (Fig 1B). Confocal images of immune-stained mosquito tissue whole-mounts revealed immunoreactive mJHBP in fat body cells and hemocytes of female adults, indicating that this is a primary site of synthesis (Figs 1C, S1C and S1E). Brightly staining foci of mJHBP accumulation were also seen in pericardial cells (Fig 1D) suggesting that the protein is being taken up pinocytically, perhaps as part of its turnover [13]. The possibility of production of mJHBP by pericardial cells cannot be ruled out, however.

### mJHBP knockout using CRISPR-Cas9: Growth and development of a mutant line

**Fig 2:**
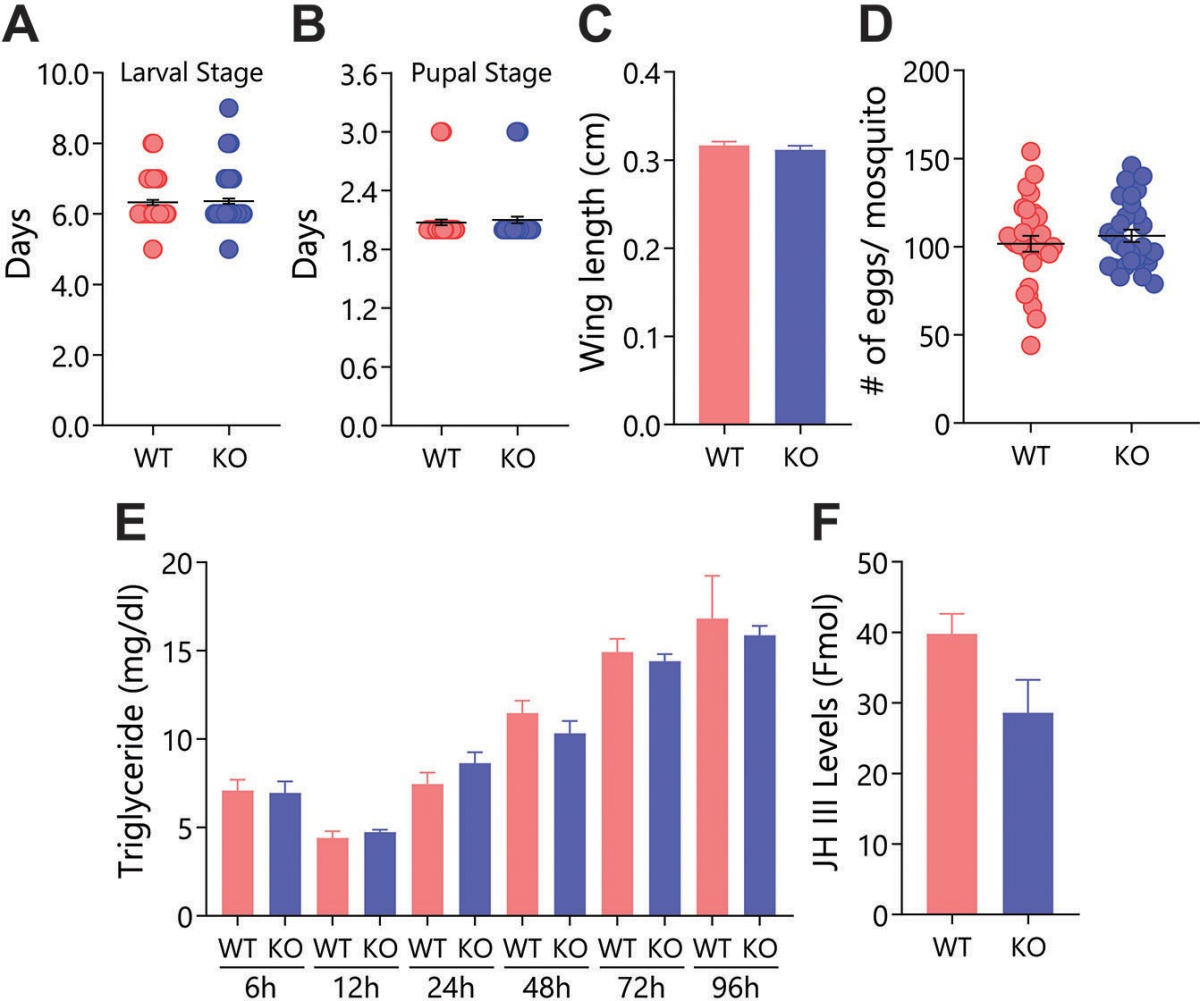
Effect of *mJHBP* deficiency on developmental and reproductive aspects in *Wild Type* (*WT*) and *mJHBP*^-/-^ (*KO*) lines. **A.** Mean time to pupation (days) from egg hatching (day 0) to the onset of pupation for *WT* and *KO* mosquitoes (4 biological replicates of 20 larvae). **B.** Mean duration of the pupal stage (days) for *WT* and *KO* mosquitoes (4 biological replicates of 20 pupae). **C.** Mean wing lengths (±SEM) for *WT* (n = 30) and *KO* (3 biological replicates of 10 adults) female mosquitoes. **D.** Mean number of eggs (±SEM) laid by *WT* and *KO* females (3 biological replicates of 9–10 adults). **E.** Quantification of triglyceride levels in *WT* and *KO* in newly emerged adult females (6 h, 12 h, 24 h, 48 h, 72 h and 96 h post adult emergence) (2 biological replicates of 2 mosquitoes for each time point). **F.** Mean JH III levels (±SE) in *WT* and *KO* mosquito hemolymph quantified by mass spectrometry (5 biological replicates, of 5 mosquitoes). None of these characteristics differed significantly (p ≤0.05) between *WT* and *KO* lines (Mann-Whitney U test).

JH plays an essential role in development and metamorphosis during the larval stage as well as in the conversion of nutritional reserves to eggs in adult mosquitoes. The absence of detectable mJHBP in larvae suggested that the protein acts in adult physiological processes such as ovarian maturation rather than larval development and metamorphosis, but this assessment is speculative. As a means of directly probing the physiological role of mJHBP, CRISPR-Cas9 methods were used to generate an mJHBP knockout line in *Ae*. *aegypti*. sgRNAs designed to target *mJHBP* (AAEL008620) at nucleotide 87 were employed to create a two-base deletion in the 5’ portion of the locus resulting in truncation of the polypeptide after proline residue 4 of the mature polypeptide. Sequencing of the pertinent genomic region confirmed that the deletion was successfully incorporated (S1A Fig). After selection of a homozygous *mJHBP* ^-/-^ line, herein referred to as the *KO* line, we were able to show loss of immunoreactive signal in hemolymph and fat body tissues using western blotting and immunofluorescence microscopy (S1B–S1D Fig). Using this mutant line, we determined its general features of growth, development and reproduction relative to wild type (*WT*) mosquitoes as an indication of function in any of these physiological systems. No dramatic differences between the *KO* and *WT* lines were seen in the duration of the larval stage, duration of the pupal stage, size, fecundity, triglyceride level or JH III titer at 24 h post-eclosion (Fig 2A–2F), indicating that mJHBP is not essential for normal development or nutrition.

### Loss of mJHBP affects innate immune defenses in response to bacterial infection

**Fig 3:**
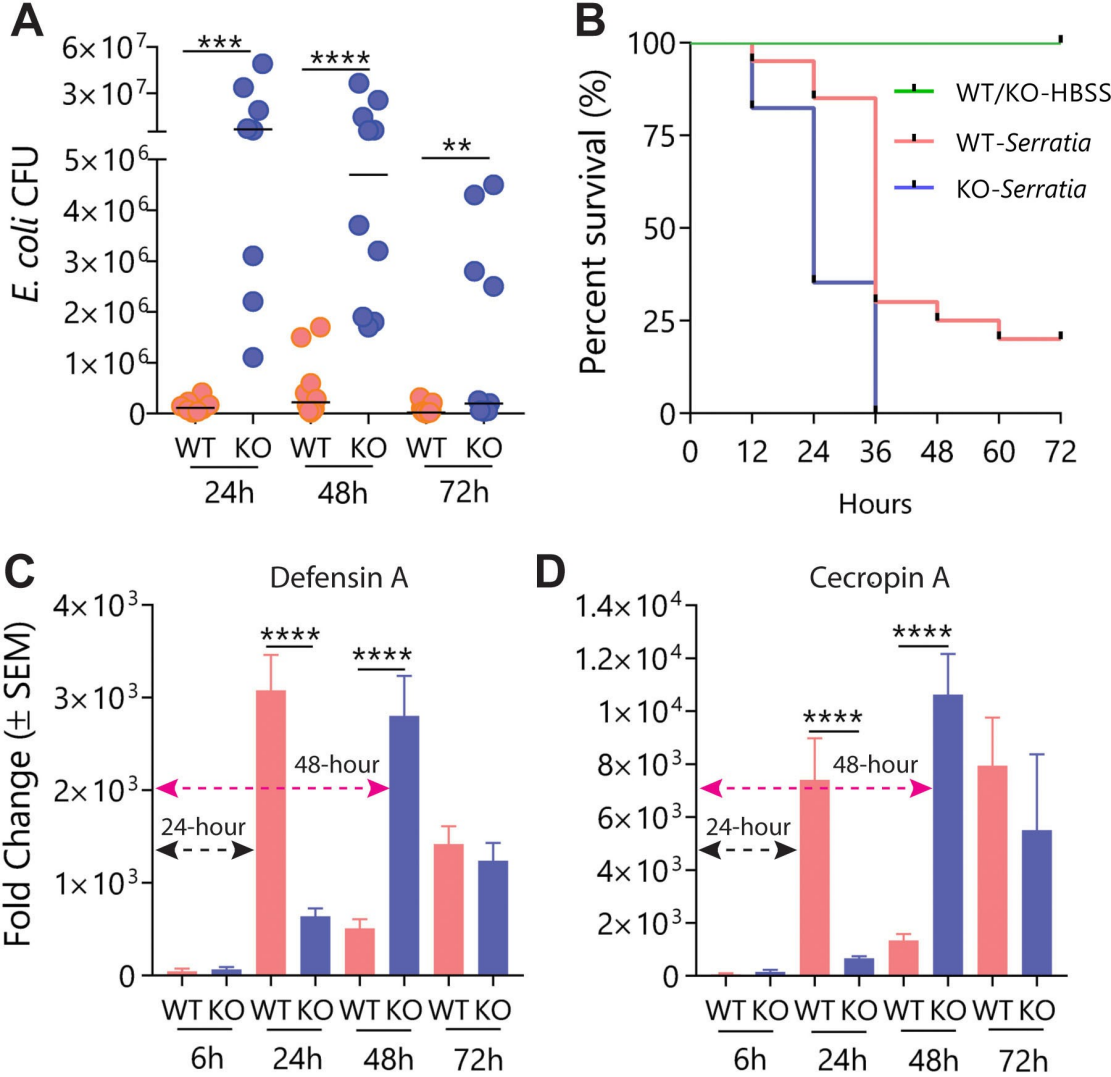
Effect of *mJHBP* deficiency on innate immune responses after bacterial challenge. **A.** *mJHBP*^-/-^ (*KO*) mosquitoes are unable to control bacterial infection. Bacterial counts (CFU) from 3 biological replicates of 2–4 individual *WT* and *KO* mosquitoes infected with *E*. *coli* (OD_600_ = 0.1) 24 (n = 8), 48 (n = 10) and 72 h (n = 11) post-infection. Significance levels (Mann-Whitney U test) are indicated as asterisks (**** p ≤ 0.0001, *** ≤ 0.001, ** ≤ 0.01, * ≤ 0.05). Median values are shown as horizontal lines. Mortality of *E*. *coli*-injected mosquitoes was negligible. **B.** Effect of mJHBP deficiency on the survival of *mJHBP*^-/-^ (*KO*) mosquitoes. Kaplan-Meier survival curves for *WT* (n = 20) and *KO* (n = 17, two biological replicates) females infected by *S*. *marcescens* (OD_600_ = 0.05) shows that KO mosquitoes succumbed earlier than *WT* females. The median survival between *WT* and *KO* mosquitoes was statistically significant (Log-rank test, p≤0.0001). **C,D.** Effect of *mJHBP* deficiency on the expression of the antimicrobial peptides *defensin A* (**C**) and *cecropin A* (**D**) in the fat body of *E*. *coli*-challenged females measured at 6, 24, 48 and 72 h post-infection (n = 9, 3 biological replicates of 3 individuals). mJHBP^-/-^ (*KO*) mosquitoes exhibited a 24 h delay in the induction of both AMPs. Changes in expression were calculated by comparing the ΔCt of infected mosquitoes with that of LB-injected mosquitoes (2^-ΔΔ^Ct). Black arrow: time of induction to maximum expression level in the *WT* strain = 24 h; Magenta arrow: time of induction to maximum expression in the *KO* strain = 48 h. Data were analyzed using the Mann-Whitney U test (**** p ≤ 0.0001). Again, mortality of *E*. *coli*-infected mosquitoes was negligible.

The general lack of effect on reproduction and development suggested that circulating mJHBP might be playing a role other than the modulation of established JH activities. Given that mosquito immunity is known to be under the control of hormonal signals, we looked for effects on antimicrobial immunity by infecting *WT* and *KO* female *Ae*. *aegypti* adults with a sub-lethal dose of an Amp^R^ *E*. *coli* strain via injection and evaluating the infection level after 24, 48 and 72 h. At 24 h post-infection, *KO* mosquitoes survived, but contained approximately 100-fold higher median bacterial levels (CFU) than *WT* mosquitoes, suggesting that mJHBP deficiency caused a reduction in the ability to fight infection (Fig 3A). After 48 h, *KO* mosquitoes continued to survive, but the bacterial counts of remained significantly higher than those of *WT* insects and were similar to the levels observed in *KO* insects at 24 h post-infection, indicating that the *KO* strain was unable to control infection by non-pathogenic bacteria for at least two days under conditions where *WT* individuals effectively limit bacterial growth (Fig 3A). At 72 h post-infection mortality remained very low, but bacterial levels in the *KO* strain dropped significantly (Fig 3A) and remained higher than those of *WT* mosquitoes, showing that induction of the immune response eventually occurred but was delayed. The same trend was observed when mortality was measured after infection with a highly pathogenic strain of *Serratia marcescens* [14]. At 24 h post infection, 35% of KO mosquitoes survived, compared with over 80% of *WT* insects (Fig 3B). At 36 h post infection, mortality in the KO strain was 100%, while 30% of infected *WT* mosquitoes survived, a difference that was statistically significant (Fig 3B).

**Fig 4:**
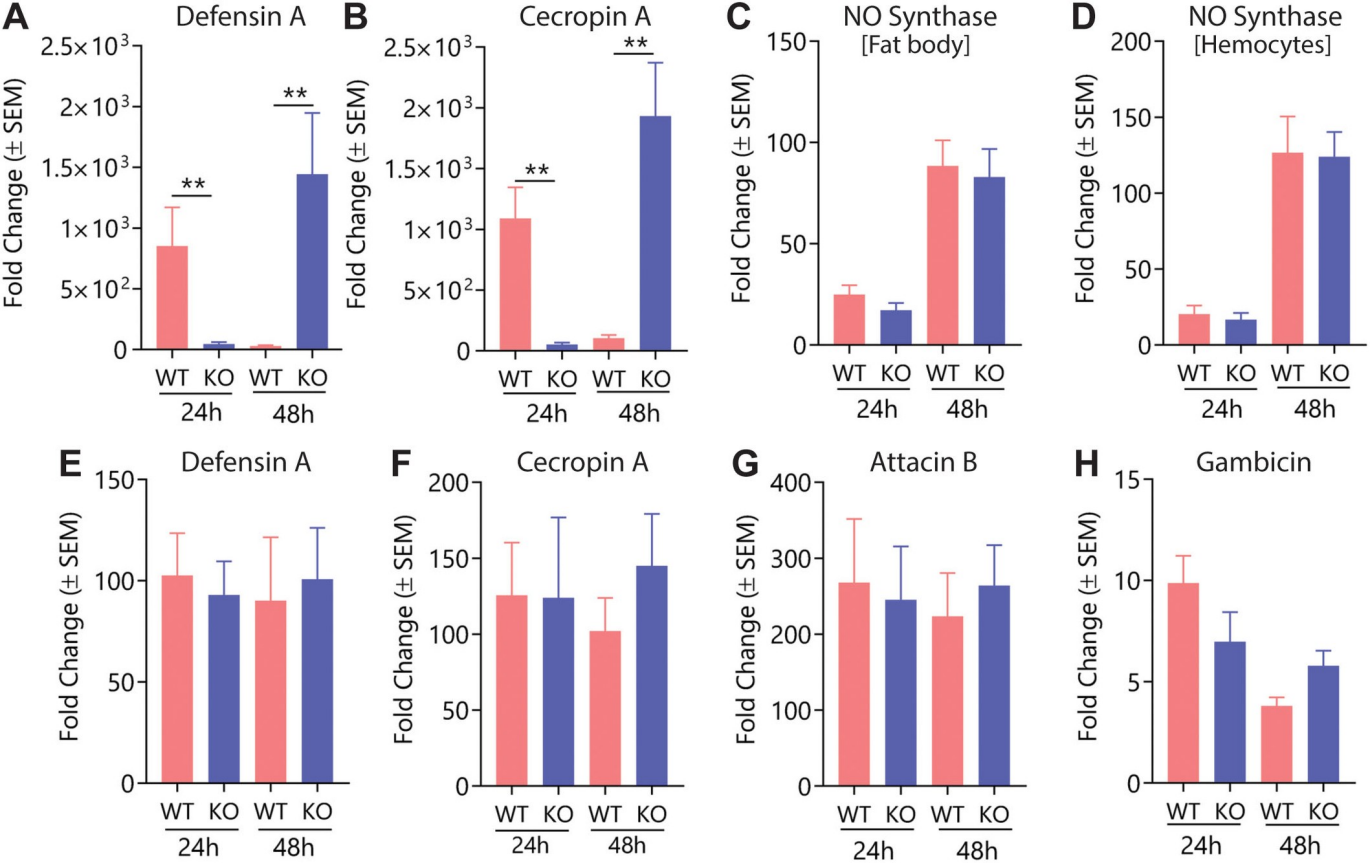
Effect of mJHBP deficiency on the expression of markers of immune function in hemocytes, fat body and midgut tissues. Relative expression levels for *defensin A* (**A**) and *cecropin A* (**B**) in hemocytes from *E*. *coli*-challenged *WT* and *KO* females measured at 24 and 48 h post-infection (n = 6, 3 biological replicates of 2 individuals). Relative expression levels for *nitric oxide synthase* (NOS) in fat body (**C**) and hemocytes (**D**) from *E*. *coli*- challenged females measured at 24 and 48 h post-infection (n = 6, 3 biological replicates of two individuals). Relative expression levels for *defensin A* (**E**), *cecropin A* (**F**), *attacin B* (**G**), and *gambicin* (**H**) in midguts from *E*. *coli*-challenged *WT* and *KO* females measured at 24 and 48 h post-infection (n = 6, 3 biological replicates of 2 individuals). Data were analyzed using the Mann-Whitney U test (** p ≤ 0.01).

To continue dissecting the role of mJHBP in immunity, we studied the inducibility of AMP transcription in response to *E*. *coli* infection. At 24 h post-infection, fat bodies from *WT* female mosquitoes showed 3,078- and 7,404-fold increases in transcript levels of *defensin A* and *cecropin A*, respectively, relative to mock-infected insects, while fat bodies from infected *KO* mosquitoes showed only 637- and 664-fold increases in the in the transcript levels of the same AMPs (Fig 3C and 3D). At 48 h post-infection, AMP transcript levels in *KO* females rose dramatically to levels comparable to those reached by *WT* mosquitoes at 24 h (Fig 3C and 3D, black arrows), while the expression levels in *WT* mosquitoes decreased (Fig 3C and 3D). These results clearly indicate the existence of a ~48 h delay in the activation of immune responses. A similar pattern of expression was seen in hemocytes, where *WT* mosquitoes showed higher induction levels of AMP expression (relative to mock-infected mosquitoes) at 24 h, while at 48 h expression was higher (50- and 20-fold higher for *defensin A* and *cecropin A*, respectively) in the *KO* line than in *WT* hemocytes, which contained lower levels of transcript relative to the 24 h timepoint (Fig 4A and 4B). Unlike the AMPs, expression of nitric oxide (NO) synthase in fat body and hemocytes did not differ significantly between infected *WT* and *KO* mosquitoes at the 24 h timepoint (Fig 4C and 4D). Given the importance of the midgut as an immunocompetent tissue we quantified the expression of AMP genes in this tissue in *WT* and *KO* mosquitoes in response to bacterial injection. Expression levels of *defensin A*, *cecropin A*, *attacin B* and *gambicin* were far lower in the midgut than the fat body suggesting that bacteria introduced by thoracic injection do not induce expression in midgut tissue as efficiently as in the fat body (Fig 4E–4H). The levels of AMP expression in midguts also did not differ significantly between *WT* and *KO* mosquitoes, suggesting that mJHBP may not play a role in the activation of midgut immunity after injection of bacteria into the hemocoel (Fig 4E–4H).

### mJHBP mutants differ in their ability to bind JH

**Fig 5:**
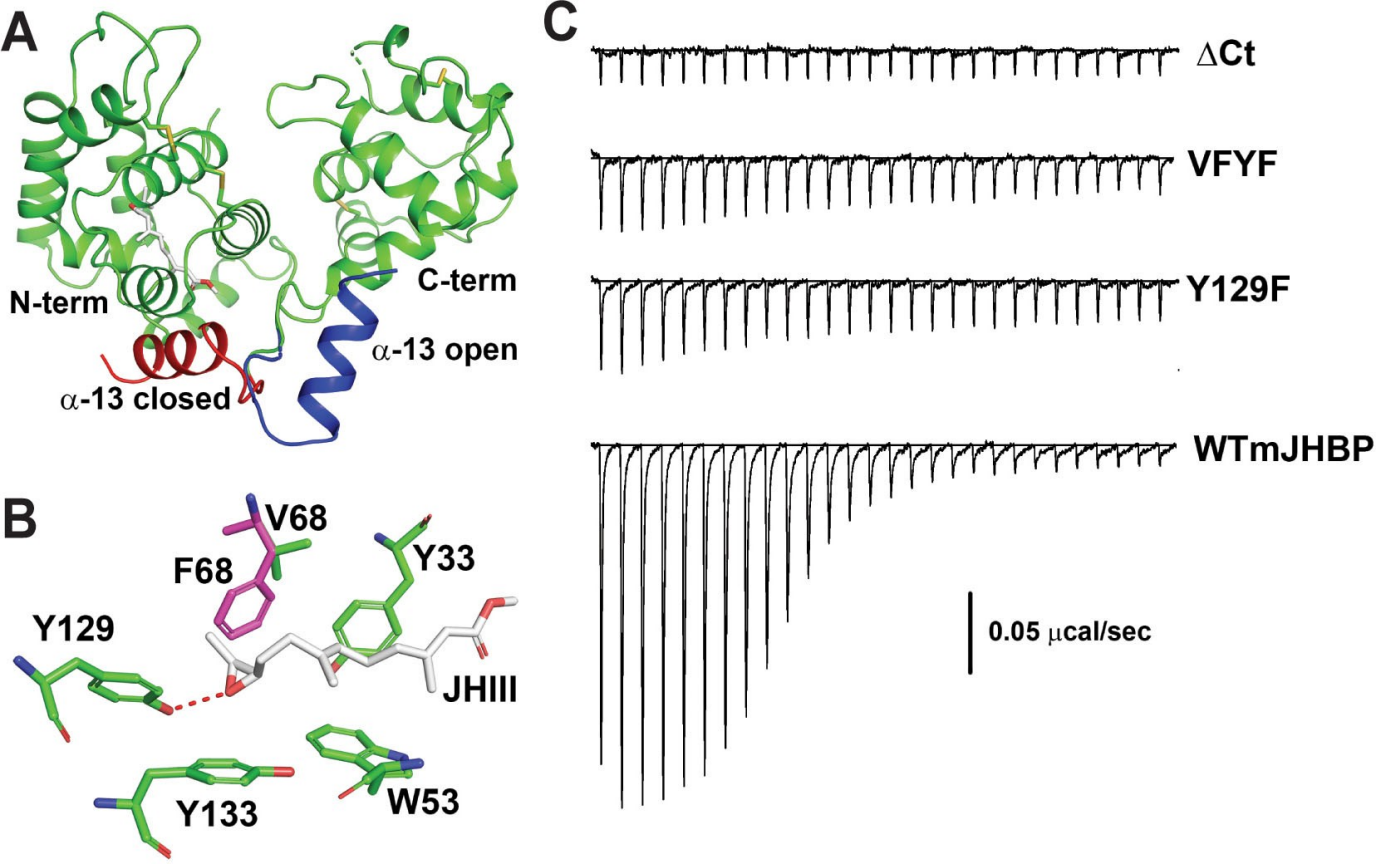
Mutagenesis and ligand binding profiling of mJHBP. **A.** The JH III binding site is located in the N-terminal domain (N-term). In the wild type mJHBP-JH III complex helix α-13 of the C-terminal domain covers the ligand access channel (red). The α-13 position in the presumed open form of the ligand-free protein would be folded back over the C-terminal domain (blue). In the ΔCt mutant α-13 is missing, apparently destabilizing the mJHBP-JH III complex. **B.** Binding site detail showing hydrogen bonding between the Tyr129 hydroxyl group and the JH III epoxy group. This residue is mutated to phenylalanine in the Y129F and VFYF mutants. In the VFYF mutant Val68 is also mutated to phenylalanine (magenta), further occluding the binding pocket. **C.** Three site-directed mutants of mJHBP were evaluated for interaction with JH III using isothermal titration calorimetry (ITC). Traces showing heats of interaction reveal highly enthalpic binding of JH III with the *WT* protein. Injection heats decrease with increasing modification of the protein (WT > Y129F > VFYF > ΔCt).

The crystal structure of the JH III complex of mJHBP provides a detailed view of the ligand binding mode and identifies ligand-contacting residues in the binding pocket of the N-terminal domain (Fig 5A) [9]. This information allowed us to design site-directed mutants to probe the role of JH III binding in the immunomodulatory function of the protein. The Y129F mutant has a single substitution of Tyr129 → Phe129 which results in loss of a protein-ligand hydrogen bond (Fig 5B). The VFYF mutant has the same amino acid substitution as the Y129F mutant plus the substitution Val68 → Phe68 that fills part of the binding pocket and would be expected to hinder ligand binding (Fig 5B). The ΔCt mutant contains the following substitutions, Tyr148 → Val148, Ile151 → Ala151 and Ile165 → Lys165, altering the structure at the entry of the binding pocket. Additionally, this mutant is truncated at position Phe269, thereby removing the C-terminal α-helical segment that caps the binding site and most-likely stabilizes the ligand-bound form of the protein (Fig 5A and 5B).

All three mutant proteins showed reduced affinity for JH III relative to the wild type protein (Figs 5C, S3 and S4). The Y129F mutant showed an approximately 10-50-fold increase in the dissociation constant (Kd) and a large reduction in the negative magnitude for the binding enthalpy (ΔH), presumably due to loss of the stabilizing hydrogen bond. The VFYF mutant exhibited an additional loss of binding affinity and further diminished binding enthalpy, while the ΔCt mutant exhibited little or no detectable interaction using calorimetric methods (Figs 5C and S4).

### Injection of recombinant mJHBP rescues the antibacterial response in *KO* females

**Fig 6:**
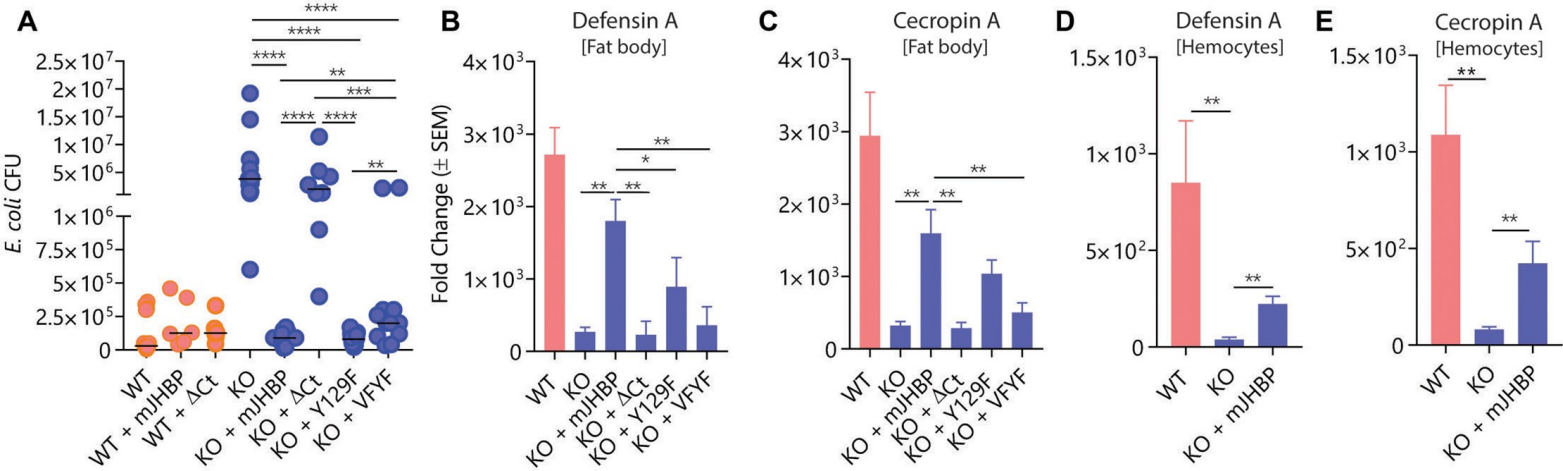
Effect of wild type and mutant mJHBP protein injection on bacterial replication and induction of antimicrobial peptides in fat bodies and hemocytes from *E*. *coli*-challenged females. Data shown in **A-C** compares the activity of wild type mJHBP protein and three JH-binding deficient mutants. **A.** The ability of mJHBP site-directed mutants to rescue the ability of *mJHBP*^-/-^ (*KO*) mosquitoes to control bacterial infection correlates with JH binding capability measured in ITC experiments. The order of immunomodulatory potency for the proteins is: WT > Y129F > VFYF > ΔCt. The responses of infected *WT* females are shown for comparison. Statistical comparisons are shown between buffer-injected females and females injected with WT mJHBP or its mutants. Significance levels (Mann-Whitney U test) are indicated with asterisks (**** p ≤ 0.0001, *** ≤ 0.001, ** ≤ 0.01). Sample sizes: *WT* (n = 13, 4 biological replicates), *WT*+mJHBP (n = 6, 3 replicates), *WT*+ΔCt (n = 6, 3 replicates), *KO* (n = 11, 3 replicates), *KO*+mJHBP (n = 12, 4 replicates), *KO*+ΔCt (n = 8, 3 replicates), *KO*+Y129F (n = 15, 5 replicates), *KO*+YFVF (n = 12, 4 replicates). Median values are shown as horizontal lines. The injection of biologically active mJHBP into *mJHBP*^-/-^ (*KO*) mosquitoes rescues the early induction of the AMPs *defensin A* (**B**) and *cecropin A* (**C**) in the fat body at 24 h after bacterial infection. AMP expression in protein-injected, *E*. *coli*-infected mosquitoes was determined relative to protein-injected, HBSS-injected (no infection) individuals by quantitative PCR using the 2^-ΔΔCt^ method. Site-directed mutant proteins vary in effectiveness in restoring AMP production corresponding to their JH III binding affinity (WT > Y129F > VFYF > ΔCt). The response of infected WT females is shown for comparison. Sample sizes: *WT* (n = 6, 3 biological replicates), *KO* (n = 6, 3 replicates), *KO*+mJHBP (n = 7, 3 replicates), *KO*+ΔCt (n = 6, 3 replicates), *KO*+Y129F (n = 6, 3 replicates), *KO*+VFYF (n = 6, 3 replicates). Treatments were compared using the Mann-Whitney U test (** p ≤ 0.01, * ≤ 0.05). The injection of biologically active mJHBP into *KO* mosquitoes rescues the early induction of the antimicrobial peptides *defensin A* (**D**) and *cecropin A* (**E**) in hemocytes at 24 h after bacterial infection. Sample sizes: (n = 6, 3 biological replicates). Data were analyzed using the Mann-Whitney U test (** p ≤ 0.01).

Since mJHBP is known to be localized to the extracellular fraction of hemolymph we hypothesized that transfer of the protein back into *KO* mosquitoes might reconstitute the *WT* immune response phenotype. Injection of recombinant mJHBP 72 h prior to infection had no significant effect on antibacterial responses of *WT* female mosquitoes 24 h post-infection, but injection into *KO* females reduced whole-body bacterial counts by 70-fold (Fig 6A). This demonstrates that the presence of mJHBP in adult-stage hemolymph is sufficient to restore a nearly wild type antibacterial response to the *KO* line. Accordingly, fat body transcript levels of the AMPs defensin A and cecropin A were also substantially increased at 24 h post-infection in mJHBP-injected, *E*. *coli*-infected *KO* mosquitoes to a level that was significantly greater than those seen in *E*. *coli*-infected, buffer-injected *KO* females (Fig 6B and 6C). Hemocytes collected from mJHBP-injected, infected *KO* females also exhibited elevated levels of AMPs relative to infected mock-injected *KO* individuals indicating a response similar to that of the fat body (Fig 6D and 6E).

Like the *WT* mosquitoes, 20–25% of *KO* individuals infected with *S*. *marcescens* after injection with mJHBP or buffer, survived beyond 72 h post-infection, while all buffer-injected mosquitoes succumbed by 48 h (S5 Fig). The survival of buffer-injected/*S*. *marcescens*-infected *KO* mosquitoes was significantly lower than infected *WT* mosquitoes (p ≤ 0.0001, Figs 3B and S5), while survival of mJHBP-injected/infected individuals was not (p = 0.2240, Figs 3B and S5), suggesting a protective effect of injected mJHBP. However, the difference in survival between buffer- and mJHBP-injected/*S*.*marcescens*-infected *KO* mosquitoes, while trending toward protection by mJHBP, did not reach the level of significance (p = 0.0841, S4 Fig), suggesting that protein-injected *KO* mosquitoes may be weakened by the injection prior to infection and die more quickly than their uninjected counterparts (Fig 3B). Due to non-proportional hazards present in the buffer-injected / mJHBP-injected comparison, we also analyzed the data by fitting an accelerated time failure model with a Weibull distribution as chosen by lowest value for Akaike’s Information Criteria (AIC) [15]. While median survival times did not differ in these groups, the time to event ratio (ETR) was significantly higher in the mJHBP-injected group compared to the buffer-injected group (ETR 1.76, p < 0.001). This indicates an overall higher survival level in the mJHBP-injected, *S*. *marcescens*-infected mosquitoes relative to buffer-injected mosquitoes.

To investigate the potential role of JH III binding by mJHBP in its immunomodulatory activity, we injected the mutant proteins into adult *KO* mosquitoes 72 h prior to *E*. *coli* infection and evaluated their effects on antimicrobial immunity relative to injection of wild type mJHBP. Females injected with the Y129F protein were able to control bacterial infection at 24 h and exhibited bacterial counts that were similar to those injected with wild type protein, indicating a retention of activity (Fig 6A). However, expression levels for the AMPs *defensin A* and *cecropin A* were approximately two-fold lower relative to *WT* mJHBP-injected females, suggesting that reduced ligand affinity diminishes the immunostimulatory activity of the protein (Fig 6B and 6C). Injection of the double mutant, VFYF, resulted in significantly higher infection levels than those observed in Y129F-injected and wild type mJHBP-injected females, but significantly lower levels than mock-injected *KO* females (Fig 6A). *Defensin A* and *cecropin A* induction were also significantly lower than in *WT* mJHBP-injected *KO* mosquitoes indicating that the protein retained some function, but was impaired (Fig 6B and 6C). Finally, injection of ΔCt, the most heavily modified mutant protein, produced bacterial counts that were not significantly lower than mock-injected *KO* mosquitoes, and resulted in AMP transcript levels that were similar to those seen in the mock-injected mutant indicating that this protein had lost most, if not all, of its activity (Fig 6A–6C). The persistence of injected *WT* mJHBP and ΔCt in the hemolymph was examined by western blotting of whole mosquito homogenates produced 48 h after protein injection (S2B Fig). Levels of the two proteins were approximately equal at this point, indicating that the lack of function in the ΔCt mutant was not due accelerated protein degradation. Hemocytes collected from mJHBP-injected, *E*. *coli*-infected *KO* females also exhibited elevated levels of AMPs relative to infected mock-injected *KO* females indicating a response similar to that of the fat body (Fig 6D and 6E). We conclude from these experiments that modification of the binding site and the consequent diminution of ligand affinity reduces the effectiveness of the protein as an immunomodulator.

### *Ae*. *aegypti KO* females are abnormal in their hemocyte composition

**Fig 7:**
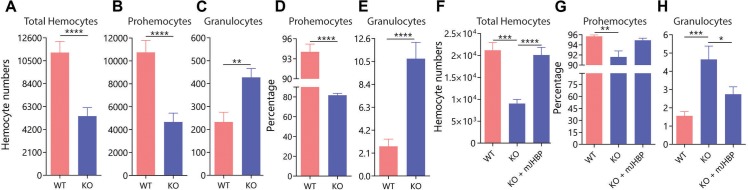
mJHBP-deficient mosquitoes exhibit impaired hemocyte development (lower hemocyte counts) and increased granulocyte differentiation. Relative abundance (Mean number ± SEM) of total hemocytes (**A**), prohemocytes (**B**) and granulocytes (**C**) per female in newly-emerged *WT* and *KO* lines. Relative proportion (%) of prohemocyte (**D**) and granulocyte (**E**) subpopulations in in newly emerged *WT* and *KO* lines. Relative abundance (Mean number ± SEM) of total hemocytes (**F**), as well as the proportions of prohemocytes (**G**), and granulocytes (**H**) perfused from newly emerged *WT* and *KO* adults reared to eclosion after injection of fourth instar larvae with buffer or biologically active mJHBP. Significance levels (Mann-Whitney U) are indicated with asterisks (**** p ≤ 0.0001, *** ≤ 0.001, ** ≤ 0.01, * ≤ 0.05). In panels **A-E** two biological replicates of 10 female mosquitoes each were counted. In panels **F-H**, two biological replicates of 4–7 mosquitoes each were performed.

Circulating hemocytes are essential for initiating and maintaining normal mosquito immune responses [16–20]. To assess the impact of mJHBP deficiency on hemocytes, we evaluated the total numbers and population structure of hemocytes in newly emerged females (S6 Fig). In hemolymph of *WT* and *KO* mosquitoes, the total number of hemocytes was 2.6-fold lower in the *KO* line (Figs 7A and S6), due largely to a drop in the number of prohemocytes, which were 3.2-fold more abundant in *WT* females (Fig 7B). Conversely, granulocyte numbers were 2.2-fold higher in *KO* mosquitoes than in *WT* individuals but were far less numerous than prohemocytes (Fig 7C). Granulocytes comprised about 2% of the total number of hemocytes in *WT* females and close to 10% in *KO* individuals (Fig 7D), while prohemocytes comprised about 94% of the total in *WT* mosquitoes and 80% in *KO* individuals (Fig 7E).

We then asked whether mJHBP could rescue the development of the immune system and ontogeny of immune cells before adult emergence. To test this hypothesis, we injected wild type mJHBP protein into fourth instar *KO* larvae and evaluated the status of hemocyte populations in newly emerged adult females. As was the case with responses to bacterial infection, hemocyte numbers and population structure in protein-injected *KO* mosquitoes came to resemble those in *WT* adults (Fig 7F–7H). After injection of protein, total hemocyte numbers were not significantly different between mock-injected *WT* and protein-injected *KO* females. However, both groups contained more than two-fold more circulating hemocytes than mock-injected *KO* individuals (Fig 7F). The proportion of prohemocytes also increased to *WT* levels in protein-injected *KO* females, whereas the proportion of granulocytes decreased to a level not significantly different than mock-injected *WT* mosquitoes (Fig 7G and 7H), indicating that mJHBP is required for normal hemocyte development during pre-adult stages.

**Fig 8:**
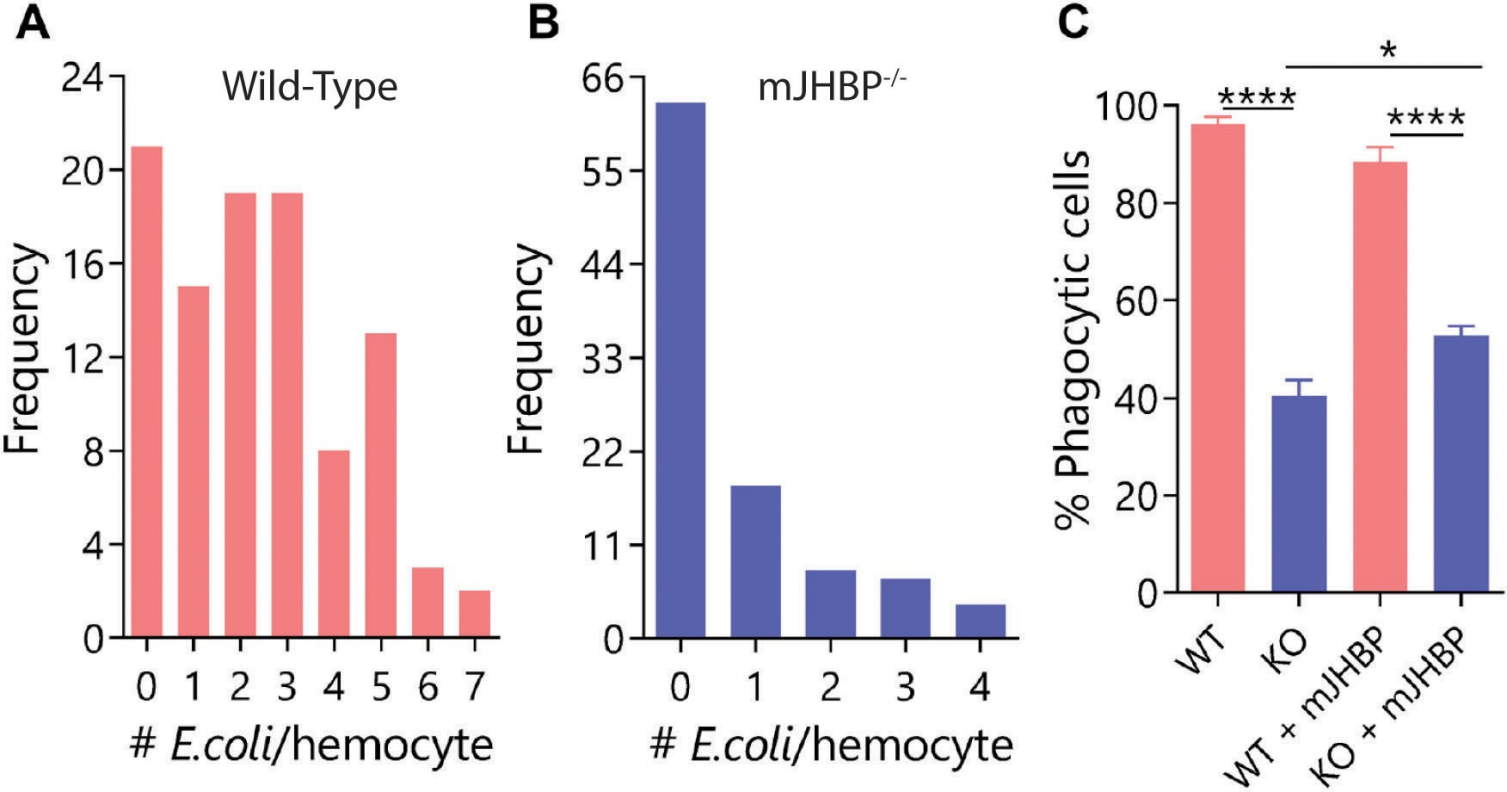
Phagocytic capabilities of hemocytes perfused from newly emerged *WT* and *KO* females. Phagocytic capacity of hemocyte from *WT* (**A**) and *KO* (**B**) lines. Data shown represents the frequency distributions of fluorescent *pHrodo E*. *coli* bioparticles per individual granulocyte (phagocyte). Two biological replicates were performed in which 10 granulocytes per mosquito from at least three fields were counted. In each replicate 5 mosquitoes were analyzed. **C**. Effect of biologically active and mutant mJHBP protein injection on the phagocytic activity (mean % ±SEM) of granulocytes in *WT* and *KO* lines. Two biological replicates were performed in which granulocytes were counted as in panels A and B. In each replicate, granulocytes from 7–12 mosquitoes were evaluated. Significance levels (Mann-Whitney U test) are indicated with asterisks (**** p ≤ 0.0001, * ≤ 0.05).

Since humoral responses appeared impaired, we hypothesized that cellular responses such as phagocytosis may also be affected. To test this hypothesis, we injected pH-sensitive fluorescent *E*. *coli* bioparticles into the hemocoel of newly emerged females and found that the *KO* line exhibited a drastic reduction in the ability to phagocytose bacteria when compared to the *WT* strain. *WT* granulocytes contained a mean of approximately 2.4 particles each while *KO* hemocytes contained only 0.7 particles 24 h post particle injection (Fig 8A and 8B). We also tested whether the presence of mJHBP changes the phagocytic activity of granulocytes by quantifying the percentage of phagocytic hemocytes present after injection of recombinant protein into newly emerged females two days prior to the injection of fluorescent *E*. *coli* particles (Fig 8C). After injection of buffer alone, *KO* granulocytes exhibited reduced phagocytic capacity where only 40% of circulating granulocytes contained *E*. *coli* particles after 1 h, while 96% of hemocytes from buffer-injected *WT* individuals contained bacterial particles at the same time point. By contrast, when mJHBP was injected (two days prior to injection of particles) into *KO* females, the phagocytic activity increased significantly (53% of granulocytes containing bacteria) when compared to buffer-injected *KO* individuals, suggesting that the wild type mJHBP was capable of partially reversing the phagocytic deficiency in the *KO* strain.

### Expression of immune response genes after bacterial challenge

**Fig 9:**
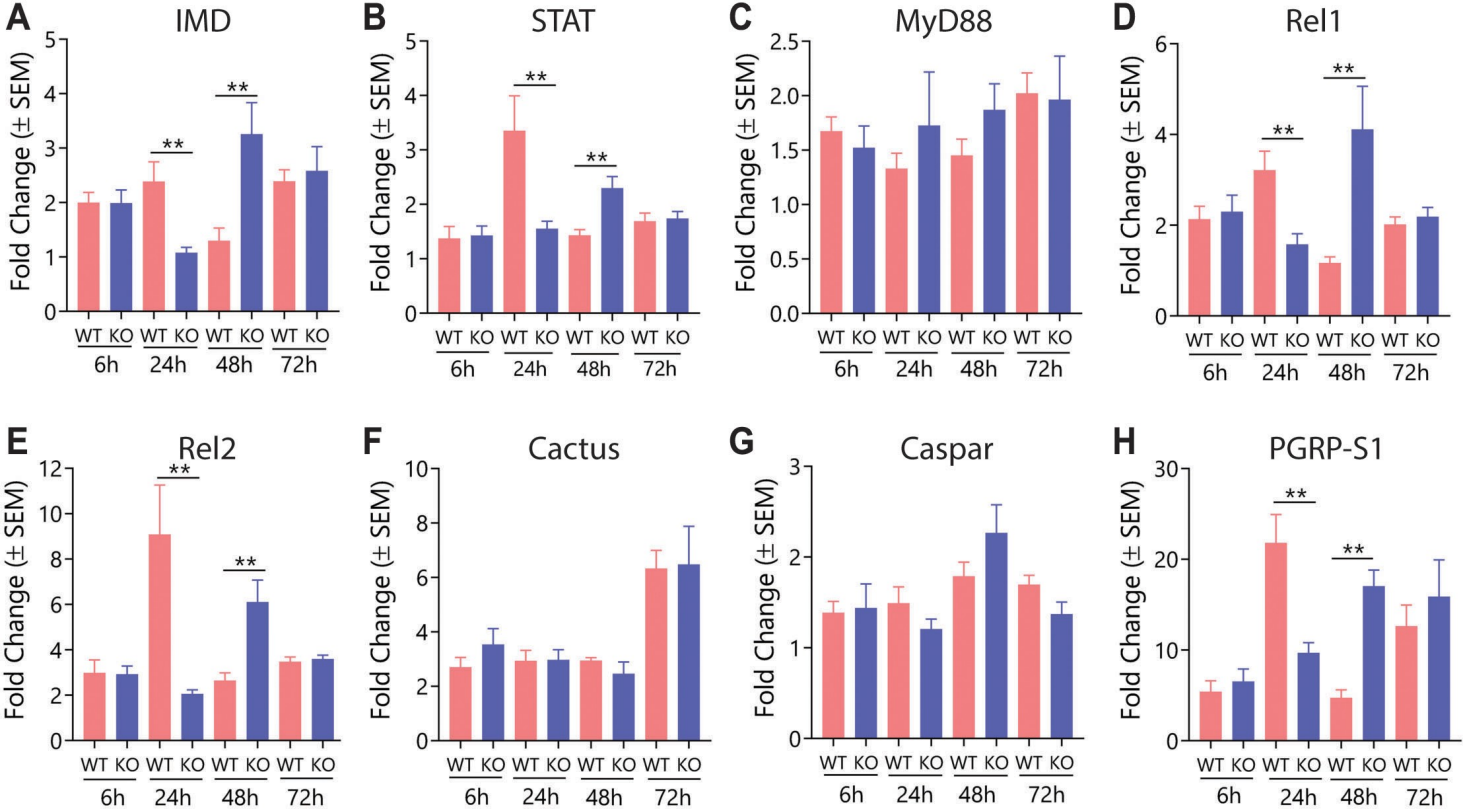
Effect of mJHBP deficiency on the expression of *Toll*, *IMD*, and *JAK/STAT* pathway components in response to bacterial challenge. Gene expression comparison between *WT* and *KO* females for: **A.** *IMD* (*IMD* Pathway); **B.** *STAT* (*JAK/STAT* pathway), **C.** *MyD88* (*Toll* pathway), **D.** *Rel1* (Toll pathway), **E.** *Rel2* (*IMD* pathway), **F.** *Cactus* (*Toll* pathway), **G.** *Caspar* (*IMD* pathway), **H.** *PGRP-S1*. Fat bodies of infected *WT* and *KO* mosquitoes were dissected at 6, 24, 48, and 72 h post-infection. Each time point represents 50 mosquitoes (5 biological replicates of 10 mosquitoes each). Significance levels (Mann-Whitney) are indicated with asterisks (p ** ≤ 0.01).

Differences in the expression of AMPs after infection in *WT* and *KO* mosquitoes indicated that the antibacterial response of the *KO* line to bacterial challenge was compromised. Because of this we decided to investigate the basis of this defect by measuring the expression of genes in the IMD, Toll and JAK/STAT pathways following *E*. *coli* infection. Transcript levels for the adaptor proteins IMD (IMD pathway) and STAT (JAK/STAT pathway) were significantly elevated in the *WT* strain 24 h after infection, while the *KO* strain showed significantly higher expression at the 48 h timepoint, echoing the expression pattern of the AMPs (Fig 9A and 9B). MyD88, an adaptor protein of the Toll pathway, was not differentially expressed at either timepoint, suggesting that primary mJHBP-mediated signaling may be operating through the IMD, or possibly the JAK/STAT pathways (Fig 9C). However, the transcript levels of the transcription factors Rel1 and Rel2 from the Toll and IMD pathways, respectively, showed significantly higher induction levels in *WT* females at 24 h and *KO* mosquitoes at 48 h suggesting that both pathways are activated on infection with *E*. *coli* in an mJHBP-dependent manner (Fig 9D and 9E). Cross induction of these pathways has previously been demonstrated and could be occurring in this case. Expression of the negative regulators *Cactus* (Toll) and *Caspa*r (IMD) did not differ between the two lines indicating that mJHBP is not acting through modulation of this type of regulation (Fig 9F and 9G). Finally, PGRP-S1, a fat body-associated peptidoglycan receptor which responds directly to bacterial stimulus, is also expressed in a pattern that reflects the expression of AMPs. Significantly higher levels of induction were seen in infected *WT* mosquitoes than in *KO* mosquitoes at 24 h post-infection, but the infected *KO* mosquitoes had a significantly larger increase at 48 h relative to *WT* mosquitoes (Fig 9H).

## Discussion

Insect immune responses are triggered by the presence of microbes or wounding in a manner that is analogous to innate immune activation in vertebrates. The modulating role of hormones or circulating hemolymph proteins is not well understood and the immense variation in insect life history characteristics may lead to idiosyncratic adaptations of these systems in different taxa. Here we describe the immunomodulatory activity of mJHBP, a mosquito-specific circulating protein that has previously been shown to bind JH III. When expression of the *mJHBP* gene was disrupted using CRISPR-Cas-9 mutagenesis, we observed a defect in the ability to fight bacterial infection characterized by a delay in the production of antimicrobial peptides, diminished expression of inducible immune pathway genes, defects in phagocytic capacity and overall inability to control bacterial replication causing increased susceptibility to infection by pathogenic bacteria such as *S*. *marcescens*. We also observed changes in the numbers and population makeup of hemocytes and an impaired ability of hemocytes to phagocytose bacterial particles in the *KO* strain. These observations are summarized in S7 Fig in relation to known changes in JH titer measured in previous studies though the adult stage [12]. Egg production in the *KO* strain is normal, and JH III titers are not dramatically reduced or elevated indicating that mJHBP is not facilitating the well-described functions of JH in ovarian development or egg production. This suggests that JH signaling through its cognate receptor, Methoprene-tolerant or Met, is operating normally in these tissues, and may not be responsible for the immunomodulatory response [21, 22]. Importantly, when recombinant mJHBP is added back to the hemolymph by injection, followed by bacterial infection, the production of antimicrobial peptides, antibacterial activity and hemocyte composition are returned to levels resembling the *WT* strain. It is noteworthy that the *KO* strain responds to infection by *E*. *coli*, but the response is delayed up to two days, suggesting that the high levels of infection seen at this point can initiate an immune response in an mJHBP-independent manner but that mJHBP is essential for responses in the early stages of infection when bacterial counts are low. The target tissue of mJHBP is not known at this point, but the reduction in hemocyte numbers seen in newly-emerged adult females suggests that it may affect hematopoiesis or hemocyte proliferation directly through the activation of signaling involved in lineage specification, survival and/or proliferation, given that injection of recombinant protein dramatically reversed the abnormal hemocyte phenotype. Interestingly, both the hemocytes and fat body tissues from mJHBP-injected females show an induction of AMPs in response to infection, leaving open the possibility that mJHBP can act directly on both tissues.

mJHBP belongs to the insect odorant-binding protein (OBP) family and contains two domains, each having a characteristic OBP fold. The C-terminal domain does not have a clearly defined internal binding site, but its terminus folds back over the N-terminal domain in the JH III complex and buries the ligand in the interior of the protein. In the mJHBP-deficient *KO* strain, JH levels are not greatly changed relative to *WT*, and the development of eggs appears to occur normally, suggesting that the primary function of mJHBP is not to stabilize JH III or protect it from enzymatic degradation, although the molar excess of mJHBP over JH III observed here makes it likely that JH III circulates largely in the protein-bound form. Additionally, mJHBP does not appear essential for the expression of trypsins needed for blood meal digestion or delivery of JH III to the ovaries, which develop normally in the presence or absence of the protein. However, JH III binding is apparently necessary for its immunomodulatory activity since site-directed mutation of key binding pocket residues reduces the potency of the injected protein in reconstituting a wild type antibacterial response. This being the case, several potential molecular mechanisms of action are possible. mJHBP could be sequestering JH III away from a specific target tissue resulting in a blockade of the immunosuppressive activity of the hormone observed in *D*. *melanogaster*. This would require that the ovaries and other JH target tissues are not dependent on this mechanism and respond to the hormone normally. Alternatively, mJHBP itself could interact with a yet to be identified receptor leading to activation of immune responses or stimulation of hematopoiesis. The mechanism would require JH III binding and might be analogous to that of the vertebrate retinol-binding protein, a lipocalin that binds retinol in the plasma and delivers it to tissues via a receptor-mediated mechanism [23]. Retinol uptake is dramatically impaired in the absence of the protein. A variation on this mechanism would be one in which JH III signaling through previously characterized pathways plays no direct role in immunomodulation but binding of the hormone is required for an activating conformational change in mJHBP.

An odorant binding protein, OBP6, has been shown to modulate hematopoiesis in the tsetse fly, a vector of African trypanosomiasis in a symbiont-dependent manner [24]. In this case, the protein acts in the larva, and is required for production of crystal cells, a hemocyte lineage involved in melanization responses. This protein has a single OBP domain, is not closely related in sequence to mJHBP and is not likely to bind JH. However, this result is further proof that proteins of this type can play physiological roles that are independent of the sensory system and may involve the binding of small molecules with regulatory functions. One such role appears to be the regulation of hematopoiesis.

As in vertebrate systems, hematopoietic development in *D*. *melanogaster* is biphasic with respect to timing and location [25–27]. Blood cells are first differentiated in the head mesoderm [28]. At later stages of embryogenesis, a hematopoietic organ called the lymph gland is formed and supports the hematopoietic process throughout subsequent larval development [29, 30]. At the end of the larval stage, the lymph gland breaks down and releases hemocytes into circulation [31, 32]. There are also reports of the existence of sessile populations of hemocytes in the larvae that can give rise to hemocytes [33, 34]. More controversial is the occurrence of hematopoiesis in the adult, although the existence of hematopoietic hubs has been reported [35]. In mosquitoes, the existence of hematopoietic organs or tissues in any stage have eluded characterization. The primary sites of hematopoiesis are not firmly identified although hemocytes are known to proliferate [16, 20], but the fact that the defect in production of hemocytes in the *KO* strain can be corrected by injection of recombinant mJHBP suggests that the protein may be acting directly on this process.

Ecdysteroids have been shown in several studies to have immunostimulatory effects in a variety of insects. While the role of JH is less clear, administration of JH III and JH analogs has been demonstrated to suppress the synthesis of antimicrobial peptides in *D*. *melanogaster* through an undetermined mechanism. The results presented here implicate JH in a system of antibacterial immunity in adult mosquitoes that is mediated by mJHBP circulating in the hemolymph, but possibly not through a conventional JH signaling pathway. We do not know if the protein targets the hemocytes, the fat body or both tissues. Communication between tissue types has been described in *D*. *melanogaster* immunity and could be occurring in this case. We are continuing to investigate mechanistic aspects of mJHBP function in immunity and hematopoiesis which is particularly compelling, considering the role of mosquitoes as vectors of parasitic and viral diseases.

## Materials and methods

### Mosquitoes

*Aedes aegypti* mosquitoes (Liverpool strain) were reared using the standard insectary conditions at 27°C and 80% relative humidity with a 12 h light/dark cycle. *Ae*. *aegypti* larvae were grown in a shallow pan containing deionized water and fed a defined daily regimen of fish food (ground TetraMin flakes). Adult mosquitoes were provided with a 10% dark corn syrup (Karo) solution *ad libitum*.

### Generation of mJHBP CRISPR-Cas9 knockout line

Synthetic guide RNAs (sgRNA) were generated using 60 nt-synthetic oligonucleotides (Upper case, underlined bases indicate those complementary to the mJHBP genomic sequence: sgRNA8620-1, gaaattaatacgactcactataggTGGACGTTGTTGCTACTGGTgttttagagctagaaa; sgRNA8620-2, gaaattaatacgactcactataggGGATCAGCCATTCCATGTGAgttttagagctagaaa; sgRNA8620-3, gaaattaatacgactcactataggTCAGCATTCTGTCCCTCACAgttttagagctagaaa) in a PCR reaction with a common reverse strand oligonucleotide (5’-aaaagcaccgactcggtgccactttttcaagttgataacggactagccttattttaacttgctatttctagctctaaaac-3’) using Pfx polymerase (Thermo Fisher Scientific) according to the manufacturer’s recommendations. Conditions to generate amplicons were 94°C, 10 s; 60°C for 30 s; 68°C for 15s and 34 cycles, with subsequent purification (Machery-Nagel PCR purification kit) prior to use in an *in vitro* transcription reaction with T7 polymerase. After purification of the single-stranded sgRNAs (Mega Clear, Thermo-Fisher Scientific), a solution containing 600 ng/μL Cas9 mRNA and 100 ng/μL of each sgRNA was injected into *Ae*. *aegypti* pre-blastoderm embryos as described [36, 37]. Injected embryos were allowed to hatch and surviving adults (G_0_) were mated in groups to individuals of the opposite gender from the parental strain. A subset of progeny (G_1_) from these crosses were analyzed by PCR directly from a piece of leg tissue followed by High-Resolution Melt Analysis (HRMA) as described previously [37]. PCR was performed using Phire Hot Start II DNA Polymerase (Thermo Fisher Scientific) in the presence of LC-Green dye using primers (mJHBPDF, 5’- ATCATGGCCCTTGCGCTATC -3’ and mJHBP-R, 5’- AGTTCGTGGTTGCCATTCTCC-3’). The resulting amplicons were melted using a Light Scanner (Biofire, Idaho Technologies) and differences in fluorescence determined. Amplicons from individual mosquitoes with melting profiles distinct from *WT* mosquitoes were purified as above and subjected to Sanger-based sequencing to identify any indels. Individuals with a confirmed deletion in exon 1 of mJHBP were crossed with the parental strain and progeny genotyped by leg PCR/HRMA each generation until at G_5_, heterozygous individuals were intercrossed to generate a homozygous loss-of-function mutant strain.

### Mosquito development and fecundity

*WT* and *KO Ae*. *aegypti* larvae were hatched in deionized water in a synchronized manner and 20 first-instar larvae from each population were selected, maintained as previously described and observed until every larva reached the pupal stage. Pupae were also observed until all mosquitoes had emerged as adults. Wing lengths of female *WT* and *KO Ae*. *aegypti* were measured as a correlate of body size. Wings were detached and placed on a glass slide, and the entire width of the wing was measured using a microscope and imaging software [38]. The fecundity of *WT* and *KO Ae*. *aegypti* mosquitoes was compared by counting the number of eggs laid by individual mosquitoes. *WT* and *KO Ae*. *aegypti* larvae were hatched separately and then reared until both mosquito populations reached the adult stage. Mated female mosquitoes were provided with a blood-meal and individually transferred into cups containing wet filter paper for oviposition.

### Juvenile hormone III and triglyceride quantification in *WT* and *KO* mosquitoes

Hemolymph JH III levels from *WT* and *KO* female *Ae*. *aeygpti* mosquitoes was quantified using previously described procedures with slight modifications. Briefly, hemolymph (5 μL) from five individuals was collected by perfusion as described above, with each pool constituting a single biological replicate. A 10 μl aliquot of deuterated JHIII analog (6.25 ppb) in acetonitrile was added to each sample before extraction with 600 μl hexane. The organic phase was dried under a stream of nitrogen and resuspended in 50 μl of acetonitrile and subjected to analysis by HPLC-MS/MS analyses [39].

Total triglycerides were quantified using the Triglyceride Colorimetric Assay Kit (Cayman Chemical) following the manufacturer’s instruction. Briefly, *WT* and *KO Ae*. *aegypti* female mosquitoes were collected at 6, 12, 24, 48, 72, 96 and 120 h after adult emergence. Pools of five mosquitoes at each time point were homogenized in 100 μl NP40 Substitute Assay Reagent (Cayman Chemical). The sample solution (10 μl) was incubated with 150 μl of diluted Enzyme Mixture solution (Cayman Chemical) and the absorbance (540 nm) was measured using a Microplate Reader.

### Detection of mJHBP in *Ae*. *aegypti* by immunoblotting

Embryos, eggs, 1^st^ through 4^th^ instar larvae (n = 20) and pupae (n = 5) were collected and homogenized separately in phosphate buffered saline (PBS). Hemolymph from female and male adults (n = 5) was collected by perfusion. Samples were reduced using NuPAGE sample reducing agent, separated by SDS-PAGE (NuPAGE) and blotted onto nitrocellulose. The membranes were incubated with rabbit anti-mJHBP IgG followed by goat anti-rabbit/alkaline phosphatase conjugated secondary antibody (S1 Fig). To ensure that transfer had occurred, sample blots were stained with Ponceau S solution (0.1% in 5% acetic acid, Sigma-Aldrich, S1 Fig).

### Quantification of mJHBP in *Ae*. *aegypti* by ELISA

Female mosquitoes were collected at 0, 24, 48 and 72 h post-emergence and individually homogenized in PBS. Pupae were also collected and homogenized in the same manner. A standard curve was generated with 15, 31, 62, and 125 ng of recombinant *Ae*. *aegypti* mJHBP protein (S8 Fig). Recombinant proteins and homogenized mosquito samples were individually placed into a 96 well plate, incubated with rabbit anti-mJHBP IgG (1:1000), washed, then incubated with goat anti-rabbit alkaline phosphatase conjugated secondary antibody (1:5000). Samples were treated with Alkaline Phosphate Yellow Liquid Substrate for ELISA (Sigma-Aldrich), and the absorbance at 405 nm was measured using a Microplate Reader to quantify mJHBP in each well (S8 Fig). Wells coated with 20 μg bovine serum albumin (BSA) were used as controls.

### Confocal microscopy

Tissues from 3 to 4-day old female *Ae*. *aegypti* were collected and prepared following the whole-mount fixation and immune-staining protocols to locate the site of mJHBP synthesis [17]. Fixed tissues were incubated with rabbit anti-mJHBP polyclonal serum and treated with Alexa Fluor 633-conjugated goat anti-rabbit IgG. Immuno-stained tissues were then counter-stained for actin with Alexa Fluor 488 phalloidin. Nuclei were stained with DAPI or Hoechst 33342.

Immuno-stained cross sections of fat body from *WT* and *KO* female *Ae*. *aegypti* mosquitoes were prepared to verify the absence of mJHBP in the knock-out mosquitoes. Formalin-fixed and paraffin-embedded fat body from WT and KO mosquitoes were prepared and sectioned using previously described methods [17]. Sectioned fat body was treated with α-mJHBP and subsequently incubated with Alexa-Fluor 633-conjugated goat anti-rabbit IgG. Immuno-stained fat body was then counter-stained with 4’,6-diamidino-2-phenylindole (DAPI) and mounted in ProLong Gold (Life Technologies). Images were taken using a Leica TCS SP5 laser scanning confocal microscope (Leica Microsystems) with a 100x oil immersion objective (numerical aperture 1.25–1.4) and equipped with photomultiplier tube/hybrid detectors. Fluorochromes were excited using a 488 nm argon laser for Alexa Fluor-488 and a HeNe 633 nm laser for Alexa Fluor-633. DAPI was excited using a 405 nm diode laser and Hoechst with an argon ion laser. Images were taken using software optimized sequential acquisition and variable z-steps. Images were processed using Leica LAS AF software (Leica Microsystems), Fiji (ImageJ) [40], Imaris 8.3 (Oxford Instruments) and Adobe Photoshop CC (Adobe Systems).

### Bacterial strains and bacterial challenge

*Serratia marscescens* [14] and ampicillin resistant (Amp^R^) *Escherichia coli* were used for bacterial challenge. An Amp^R^ *E*. *coli* strain was constructed by transformation with the plasmid pUC19. This strain was grown in LB broth containing 100 μg/mL ampicillin at 37°C overnight, centrifuged and the pellet was washed twice with Hank’s Balanced Salt Solution (HBSS) (Thermo Fisher Scientific), resuspended in HBSS and diluted to an OD_600_ of 0.1 before injection into mosquitoes. *S*. *marcescens* was grown in LB broth without antibiotics overnight, prepared in the same manner as the Amp^R^ *E*. *coli*, and diluted to an OD_600_ of 0.05 prior to injection.

*WT* or *KO* female *Ae*. *aegypti* (2–3 d post emergence) were anesthetized on ice and injected into the thorax with 69 nl of resuspended *S*. *marscescens* or Amp^R^ *E*. *coli* (~5500 *E*. *coli* cells or 2750 *S*. *marcescens* cells) using a 3.5” glass capillary needle and a Nanoject III programmable Nanoliter Injector (Drummond Scientific Company). Live mosquitoes were counted every 12 h, and Amp^R^ *E*. *coli-*infected WT and KO *Ae*. *aegypti* mosquitoes were collected at 6, 24, 48 and 72 h post infection and individually homogenized in 1 ml of Luria Bertani (LB) broth. Mosquito homogenates were serially diluted in LB broth and 100 μl from each dilution was spread onto LB-Amp agar plates. Bacterial plates were incubated overnight at 37°C and the colony-forming units (CFU) per plate were used to quantify the level of infection.

### Mosquito hemolymph and tissue collection

Hemolymph, fat body and midgut tissues from *E*. *coli*- or HBSS-injected WT and KO female mosquitoes were collected at 6, 24, 48 and 72 h post injection. Hemolymph was collected using a previously described perfusion procedure [41]. Briefly, an incision was made in the abdominal wall between the last two segments using a sterile scalpel, and 10 μl of bleeding buffer [85% Schneider’s medium, 10% fetal bovine serum (FBS), and 5% citrate buffer (pH 4.5; 98 mM NaOH, 186 mM NaCl, 1.7 mM EDTA, and 41 mM citric acid)] was gradually injected into the thorax and collected using a siliconized pipette tip; a total of 10 μL of diluted hemolymph was collected from each mosquito. For RNA extraction from hemocytes, perfusates (5 μL) were placed immediately into TRIzol. Midgut and fat body from the same mosquito were also collected using sterile fine-tip forceps, placed in ice-cold TRIzol, and transferred to liquid nitrogen until all dissections were completed, then stored at -80°C.

### RNA isolation and quantitative PCR

Total RNA from hemocyte, fat body or midgut was isolated following tissue collection by the protocol described above. RNA was extracted from each tissue in TRIzol (Life Technologies) using phase lock gel tubes (Quanta Bio), and isolated using manufacturer protocols. The concentration and quality of RNA samples was assessed using a NanoDrop spectrophotometer (Thermo Scientific) and cDNAs were synthesized from the corresponding RNA samples using the QuantiTech Reverse Transcription Kit (Qiagen). Synthesized cDNA samples were used as templates for quantitative PCR assays using DyNAmo HS SYBR green qPCR kits (Thermo Fisher Scientific) and gene-specific primers (S1 Table). Prepared qPCR mixtures were analyzed for RNA abundance using the CFX 96 Real-Time PCR Detection System (Bio-Rad) and normalized against the reference gene ribosomal protein S7 (AAEL009496, S1 Table). Collected data were analyzed using the Bio-Rad CFX Maestro software (Bio-Rad), and fold change values were calculated using the 2^-ΔΔCt^ method [42]. All reactions were run in duplicate (technical), and the gene expression differences reported were based on at least three independent experiments.

### Intrathoracic injection of mJHBP proteins followed by bacterial challenge

Purified *Ae*. *aegypti* mJHBP and mutant mJHBP proteins were diluted into PBS and injected separately into one day old *WT* and *KO* female *Ae*. *aegypti* mosquitoes at a concentration of 15 ng / 69 nL using the procedure described earlier. Injected mosquitoes were allowed to recover for 2 days, then infected with Amp^R^ *E*. *coli* as described above. Infected mosquitoes were collected 24 h post-infection to determine bacterial levels and AMP expression as described above.

### Site-directed mutagenesis of *Ae*. *aegypti* mJHBP

Site-directed mutants of mJHBP were prepared by *de novo* synthesis of cDNA (Bio Basic Inc.) or by a PCR-based procedure (primer information available in S1 Table). The mutant proteins were expressed in *E*. *coli* using the same methods as for the wild type protein. Three mutation targets were identified based on the crystal structure of the mJHBP-JH III complex. In the Y129F mutant, Tyr129 was replaced by phenylalanine to eliminate a protein-ligand hydrogen bond involving the ligand epoxy group. The YFVF mutant combined the Y129F mutation with mutation of Val68 to phenylalanine that would be expected to partially obstruct access to the binding pocket. In the third mJHBP mutant, ΔCt, Tyr148 was replaced by valine, Ile151 by alanine, and Ile165 by lysine to alter the structure at the entry to the binding pocket. Additionally, this mutant contained a premature stop codon resulting in truncation of the protein immediately N-terminal to Phe269, causing a loss of the C-terminal binding pocket cap structure.

The mutant mJHBP cDNAs were cloned into pET17b, expressed and refolded from inclusion bodies following previously described procedures [9]. Wild type mJHBP and the mutant proteins were purified by gel filtration (Sephacryl S-200 and Superdex 75, GE Healthcare), ion-exchange (Q-Sepharose, GE Healthcare), and hydrophobic interaction (Phenyl-Sepharose, GE Healthcare) chromatography as described previously. The mutant proteins were evaluated using SDS-PAGE and gel filtration chromatography on Superdex-75 (S3 Fig).

### Isothermal titration calorimetry

ITC experiments were performed using a Microcal VPITC instrument at 30°C. *Ae*. *aegypti* mJHBP and mutated mJHBP proteins (Y129F, ΔCt, and VFYF mJHBP) were prepared in TBS (20 mM Tris HCl pH 7.5, 0.15M NaCl). Calorimetry experiments were performed as described previously with a protein concentration in the calorimeter cell of 5 μM and a JH III concentration in the syringe of 50 μM. Where possible, injection enthalpies were fit to a single-site binding model using the Microcal data evaluation software.

### Hemocyte counting

Hemolymph perfusates were placed in a sterile disposable hemocytometer slide (Neubauer Improved, iNCYTO C-Chip DHC-N01) immediately after perfusion [20, 43]. For granulocytes, we counted the total number of hemocytes in five quadrants using a compound microscope at 40x magnification, and the number of granulocytes was calculated as a fraction of the total number present on each sample (S6 Fig) [20, 43]. In experiments performed to evaluate the effect of mJHBP injection, fourth instar larvae where rinsed in fresh water and cooled on ice in 20 ml of water. After 5–10 mins, these were gently transferred to a wet piece of filter paper sitting atop a glass petri dish filled with crushed ice. Larvae were pat dried using a strip of paper, then injected with 69 nL of HBSS containing 15 ng of protein under the pleura in the 3^rd^ abdominal segment distal from the tail. After injection, larvae were transferred to a paper cup filled with clean fresh water and a small amount of food was added 3 hours post injection. Control larvae were injected with HBSS alone. Both groups of larvae were allowed to develop to adulthood when hemocytes were perfused and counted as described above from newly emerged females.

### Phagocytosis assays

The phagocytic capacity of granulocytes was measured by injecting 69 nl of a solution containing pHrodo-labeled *E*. *coli* bioparticles suspended at 1 mg/ mL (~1 x 10^8^ particles) in HBSS into newly emerged females. After 24 h, hemocytes were extracted as previously described, allowed to attach to a glass slide, washed using Life Technologies Live Cell Imaging Solution (Cat No. A14291DJ) and observed in Live Cell Imaging Solution supplemented with trypan blue to quench extracellular fluorescence. The number of individual bioparticles in granulocytes was determined in randomly-selected microscopy fields using a Leica epifluorescence microscope. Phagocytic activity was also assessed using pHrodo-labeled *E*.*coli* bioparticles (Life Technologies) at the concentration indicated above. At 1 h post injection hemocytes were extracted, allowed to attach to a glass slide, washed with Live Cell Imaging Solution and observed in Live Cell Imaging Solution supplemented with trypan blue to quench extracellular fluorescence. We counted the numbers of granulocytes that had ingested red-fluorescent bacteria. We evaluated differences between *WT* and *KO* mosquitoes that had been pre-injected with 69 nL (15 ng) of mJHBP protein or buffer (HBSS) 2-days prior to the delivery of bioparticles in newly emerged females.

### Statistical analysis

All statistical analyses were conducted using GraphPad Prism 7 (GraphPad) or R. Sample sizes and statistical significances were described in figures or the corresponding figure captions.

## Supporting information

S1 Fig Verification of mJHBP^-/-^ mosquito lines (*KO*) generated by CRISPR-Cas9 methods. **A.** Sequence comparison between *WT* and *KO* mosquitoes (G12) showing the deletion of two nucleotides (TG) in the target gene (AAEL008620). Features of the sequence are marked on the figure (SPC = signal peptide cleavage site). **B.** Immunodetection of mJHBP in *WT* and *KO* female mosquitoes (G12) by western blot: 1. *WT* Females (n = 10); 2. *KO* Females (n = 10); red circle = no corresponding protein band. Molecular weights of standards are shown on left. **C.** Confocal image of a 30 μm-thick fat body tissue section from *WT* female *Ae*. *aegypti* immuno-stained with α-mJHBP antibody. Nuclei, stained with DAPI (magenta), mJHBP, green; Scale bar = 30 μM. **D.** Confocal image of a 50 μm fat body tissue cross section from *KO* female *Ae*. *aegypti* immuno-stained with an α-mJHBP antibody. Nuclei, stained with DAPI (magenta), mJHBP, green; Scale bar = 20 μM. mJHBP signal was not detected in the fat body of *KO* mosquitoes. **E.** Hemocytes from *KO* (top row) and *WT* (bottom row) females stained for nuclei (Hoechst 33342, blue), actin (phalloidin, red) and mJHBP (green), demonstrating the presence of mJHBP protein in *WT* but not *KO* hemocytes.
(TIF)

S2 Fig Detection of mJHBP protein in *Ae*. *aegypti* under various conditions. **A.** Presence of mJHBP in larval, pupal and adult wild-type *Ae*. *aegypti*. Left: Nitrocellulose filter containing transferred larval (3^rd^ instar, L), early pupal (P) and adult (female, A) homogenates representing two to three individuals and stained with Ponceau S solution to detect total protein. Right: After complete destaining in 0.1 M sodium hydroxide solution, the filter was used for immunodetection of mJHBP. Although protein is detectable in all three samples, immunoreactive mJHBP is only detectable in pupal and adult samples. **B.** Detection of *WT* mJHBP and ΔCt-mutant protein from whole body homogenates of *KO* females after injection of 69 nL of PBS containing 15 ng of each protein. Top: The samples were homogenized 48 h after injection, and 1 female equivalent was separated by SDS-PAGE and blotted to nitrocellulose. Bottom: A Coomassie blue-stained gel containing 4.5 μg of each protein from stock solutions of each protein is shown for comparison.
(TIF)

S3 Fig Chromatographic analysis of recombinant mJHBP and mutants by gel filtration on Superdex 75 (10/300) eluted with 20 mM Tris-HCl, pH 8.0, 150 mM NaCl. **A.** *Wild type*-mJHBP, **B.** *Y129F*-mutant, **C.** *VFYF*-mutant and **D.** Δ*Ct*-mutant.
(TIF)

S4 Fig Isothermal titration calorimetry (ITC) profiles for JH III binding of *mJHBP* and the site-directed mutants *Y129F*, *VFYF* and Δ*Ct*. Plotting of injection enthalpies obtained from integration of measured heats vs. molar ratio indicates progressively lower affinity binding. **A.** wild type mJHBP. Calorimeter cell protein concentration = 5 μM, syringe JH III concentration = 50 μM. Ka = 1 x 10^7^ ± 1 x 10^6^ (M^-1^), ΔH = 13.0 ± 0.2 kcal/mol. **B.** Y129F mutant. Calorimeter cell protein concentration = 5 μM, syringe JH III concentration = 50 μM. Saturable binding is observed, but heats are of low magnitude. Ka = 9.2 x 10^5^ ± 7.1 x 10^5^ (M^-1^), ΔH = 3.1 ± 2.2 kcal/mol. **C.** VFYF mutant. saturable binding is weak but detectable. **D.** ΔCt mutant. Little concentration-dependent change in the heats of interaction were detected.
(TIF)

S5 Fig Effect of mJHBP injection on the survival of *mJHBP*^-/-^ (*KO*) mosquitoes infected by *Serratia marcescens*. Kaplan-Meier survival curves for *WT* (n = 20) and *KO* (n = 17, two biological replicates) females infected by *S*. *marcescens* (OD_600_ = 0.05) two days after injection with 15 ng of mJHBP in 69 nL or 69 nL of PBS. The data were analyzed using the log-rank test as well as a fit to an accelerated time failure model with a Weibull distribution [15]. The results are described in the text.
(TIF)

S6 Fig Morphology and numbers of hemocytes in *WT* and mJHBP-deficient mosquitoes. **A.** Confocal images of *Ae*. *aegypti* hemocytes stained with phalloidin (green) and Hoechst 33342 (nuclei, blue). Three distinct morphologies were identified: prohemocytes were small and showed no spreading, oenocytoids were circular in shape, larger and showed no spreading, while granulocytes were also large but showed spreading and contained visible inclusions. Prohemocytes have a higher nuclear to cytoplasmic ratio compared to oenocytoids. Maximum projection image. Scale bar = 5 μm. **B.** Low magnification bright field images of hemocytes in a hemocytometer collected from *WT* and *KO* mosquitoes by perfusion as described in the Materials and Methods. Black arrows indicate prohemocytes and blue arrows, granulocytes. Scale bar = 110 μm.
(TIF)

S7 Fig Schematic model of proposed effects of mJHBP in relation to known JH titers in *Ae*. *aegypti*. In the *KO* mutant, low hemocyte numbers and an altered hemocyte population structure lead to weak initial immune responses to bacterial infection and increased susceptibility to bacterial pathogens. JH levels are low in the pupal stage when mJHBP expression is initiated, but after the adult molt they increase dramatically and function in the previtellogenic development of the ovary. At this point mJHBP would be loaded with JHIII which may trigger stimulation of hematopoiesis and immunity. We know that injection of recombinant protein into the late larval stage or the adult stage (marked in diagram) rescues either normal hemocyte development or a normal antibacterial immune response, or both. Experiments with site-directed mutants suggest that JH binding may be essential for mJHBP function in these systems.
(TIF)

S8 Fig **A.** Standard curve used for the mJHBP quantifications by ELISA. Recombinant mJHBP was diluted to final concentrations of 125, 62, 31, and 15 ng and the OD_405_ of each concentration was measured as described in the Materials and Methods. **B.** Comparison of ELISA signal from control (BSA alone, 15 μg), larval homogenate (16 μg protein), adult homogenate (13 μg). The signal from larval homogenates was similar to the control.
(TIF)

S1 Table Primers used in this study. (DOCX)
