# Supplementary figures and images for: A mosquito juvenile hormone binding protein (mJHBP) regulates the activation of innate immune defenses and hemocyte development
Source: PLoS Pathog. 2020 Jan 21;16(1):e1008288. doi: 10.1371/journal.ppat.1008288 (PMC6994123; doi:10.1371/journal.ppat.1008288)

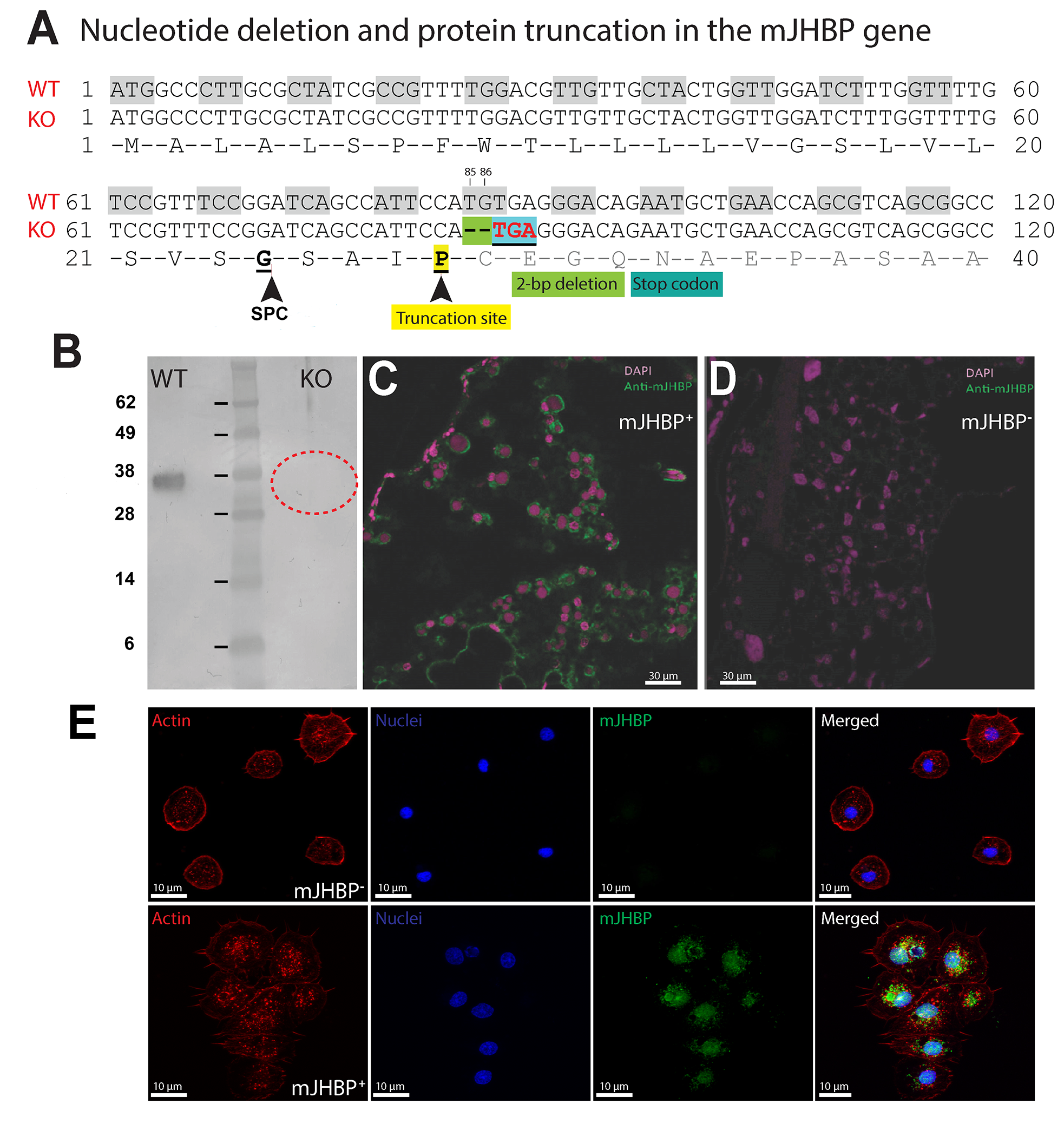

Supplement: S1 Fig — A. Sequence comparison between WT and KO mosquitoes (G12) showing the deletion of two nucleotides (TG) in the target gene (AAEL008620). Features of the sequence are marked on the figure (SPC = signal peptide cleavage site). B. Immunodetection of mJHBP in WT and KO female mosquitoes (G12) by western blot: 1. WT Females (n = 10); 2. KO Females (n = 10); red circle = no corresponding protein band. Molecular weights of standards are shown on left. C. Confocal image of a 30 μm-thick fat body tissue section from WT female Ae. aegypti immuno-stained with α-mJHBP antibody. Nuclei, stained with DAPI (magenta), mJHBP, green; Scale bar = 30 μM. D. Confocal image of a 50 μm fat body tissue cross section from KO female Ae. aegypti immuno-stained with an α-mJHBP antibody. Nuclei, stained with DAPI (magenta), mJHBP, green; Scale bar = 20 μM. mJHBP signal was not detected in the fat body of KO mosquitoes. E. Hemocytes from KO (top row) and WT (bottom row) females stained for nuclei (Hoechst 33342, blue), actin (phalloidin, red) and mJHBP (green), demonstrating the presence of mJHBP protein in WT but not KO hemocytes. (TIF) [file ppat.1008288.s001.tif]

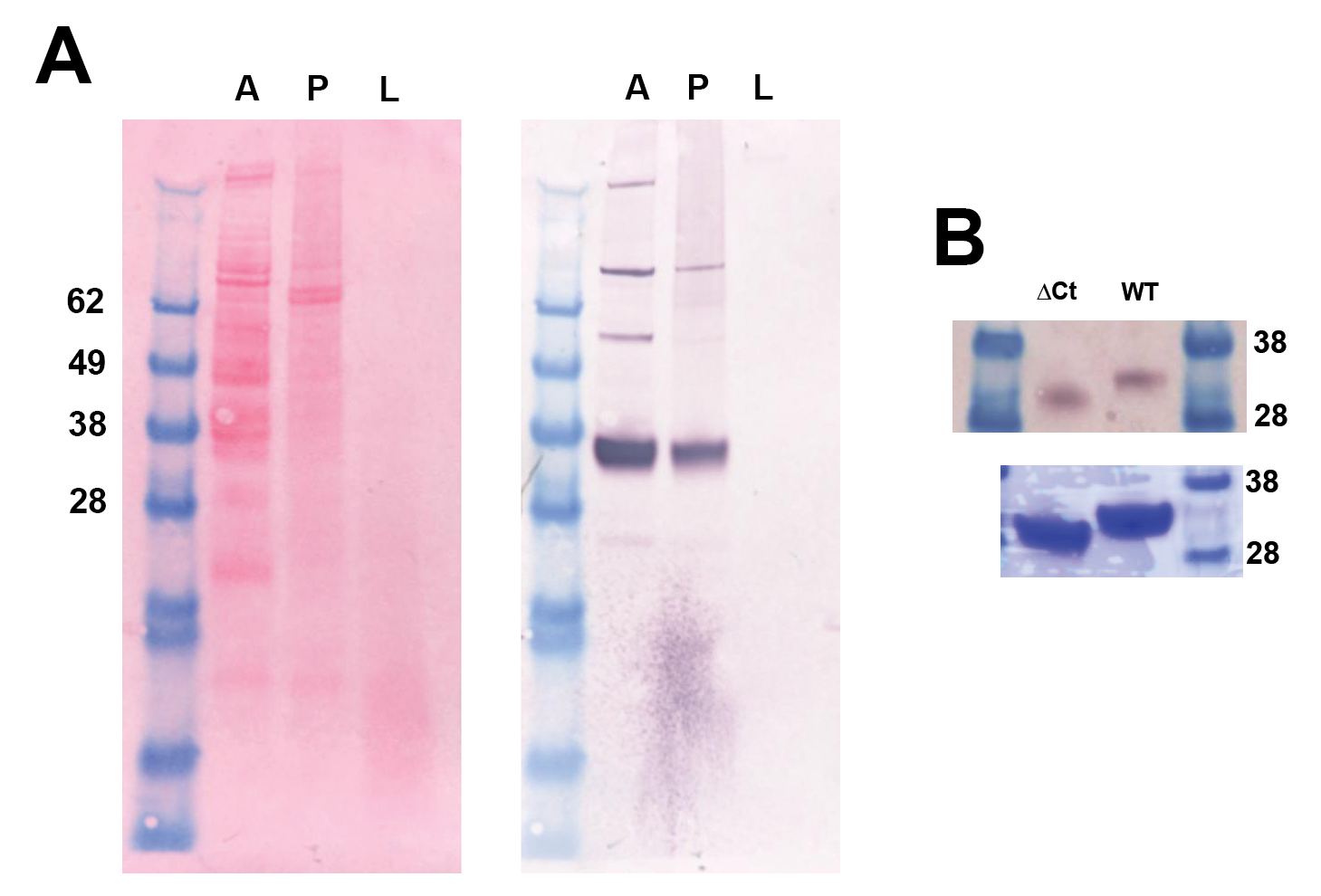

Supplement: S2 Fig — A. Presence of mJHBP in larval, pupal and adult wild-type Ae. aegypti. Left: Nitrocellulose filter containing transferred larval (3rd instar, L), early pupal (P) and adult (female, A) homogenates representing two to three individuals and stained with Ponceau S solution to detect total protein. Right: After complete destaining in 0.1 M sodium hydroxide solution, the filter was used for immunodetection of mJHBP. Although protein is detectable in all three samples, immunoreactive mJHBP is only detectable in pupal and adult samples. B. Detection of WT mJHBP and ΔCt-mutant protein from whole body homogenates of KO females after injection of 69 nL of PBS containing 15 ng of each protein. Top: The samples were homogenized 48 h after injection, and 1 female equivalent was separated by SDS-PAGE and blotted to nitrocellulose. Bottom: A Coomassie blue-stained gel containing 4.5 μg of each protein from stock solutions of each protein is shown for comparison. (TIF) [file ppat.1008288.s002.tif]

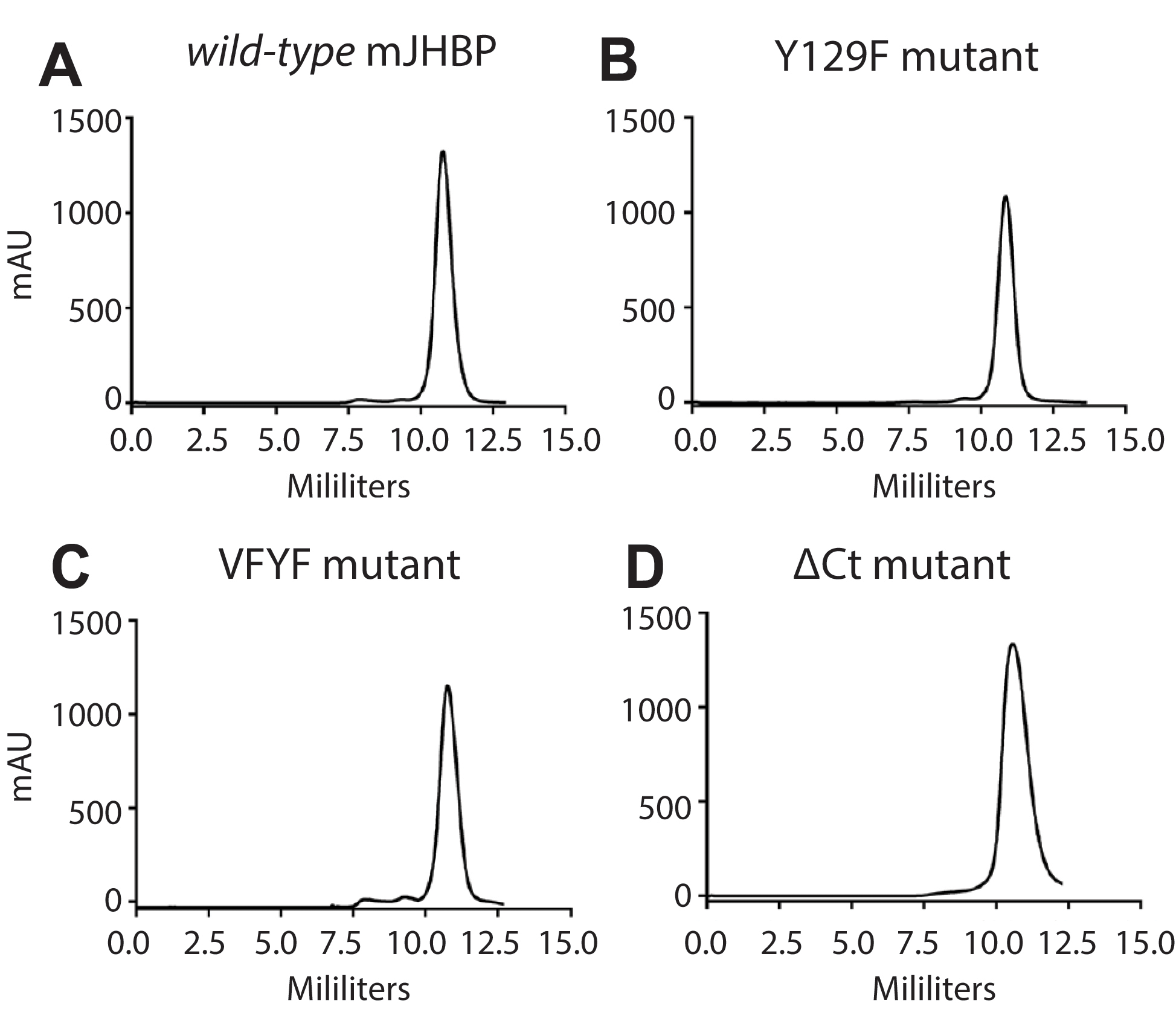

Supplement: S3 Fig — A. Wild type-mJHBP, B. Y129F-mutant, C. VFYF-mutant and D. ΔCt-mutant. (TIF) [file ppat.1008288.s003.tif]

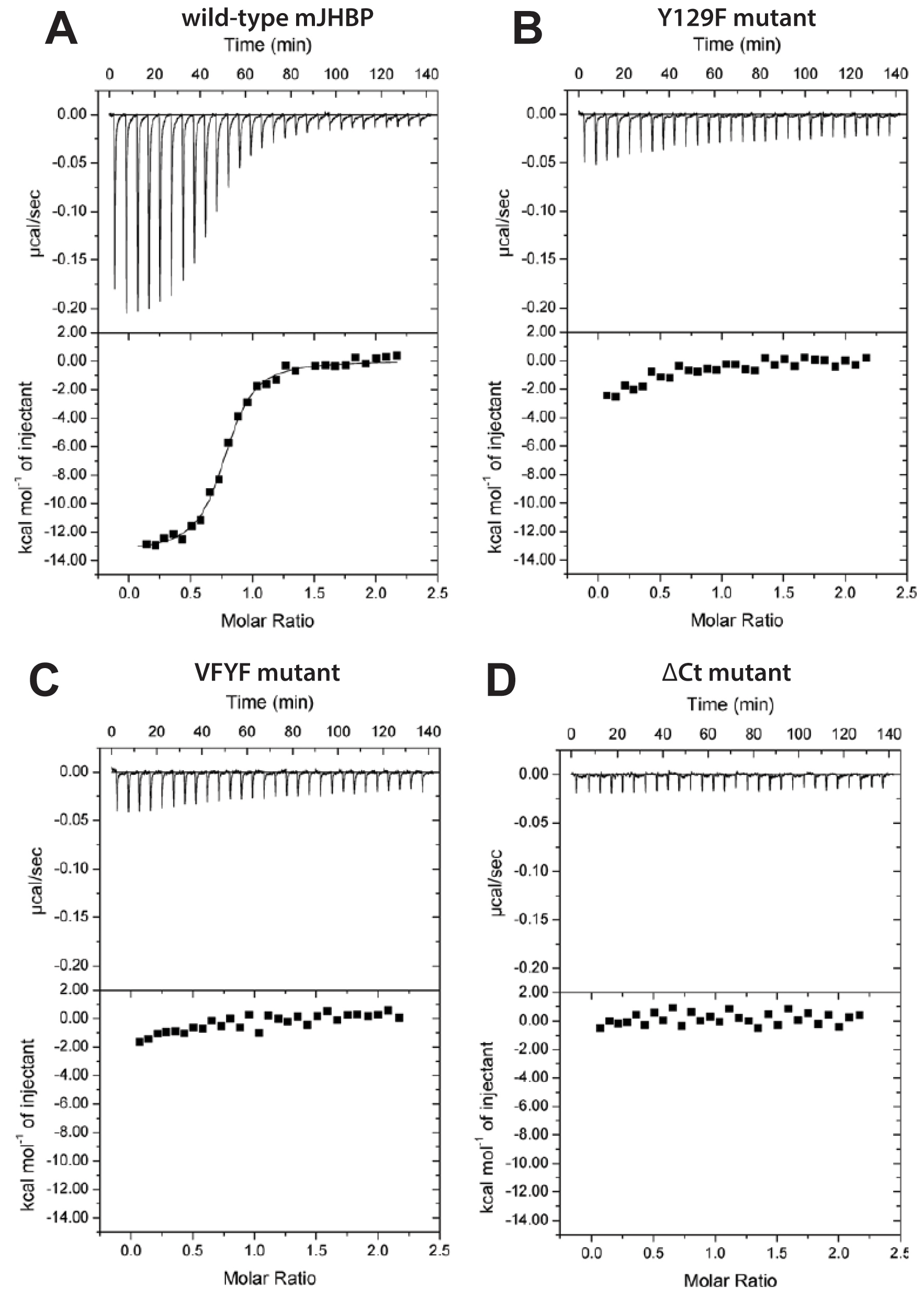

Supplement: S4 Fig — Plotting of injection enthalpies obtained from integration of measured heats vs. molar ratio indicates progressively lower affinity binding. A. wild type mJHBP. Calorimeter cell protein concentration = 5 μM, syringe JH III concentration = 50 μM. Ka = 1 x 10^7^ ± 1 x 10^6^ (M-1), ΔH = 13.0 ± 0.2 kcal/mol. B. Y129F mutant. Calorimeter cell protein concentration = 5 μM, syringe JH III concentration = 50 μM. Saturable binding is observed, but heats are of low magnitude. Ka = 9.2 x 10^5^ ± 7.1 x 10^5^ (M-1), ΔH = 3.1 ± 2.2 kcal/mol. C. VFYF mutant. saturable binding is weak but detectable. D. ΔCt mutant. Little concentration-dependent change in the heats of interaction were detected. (TIF) [file ppat.1008288.s004.tif]

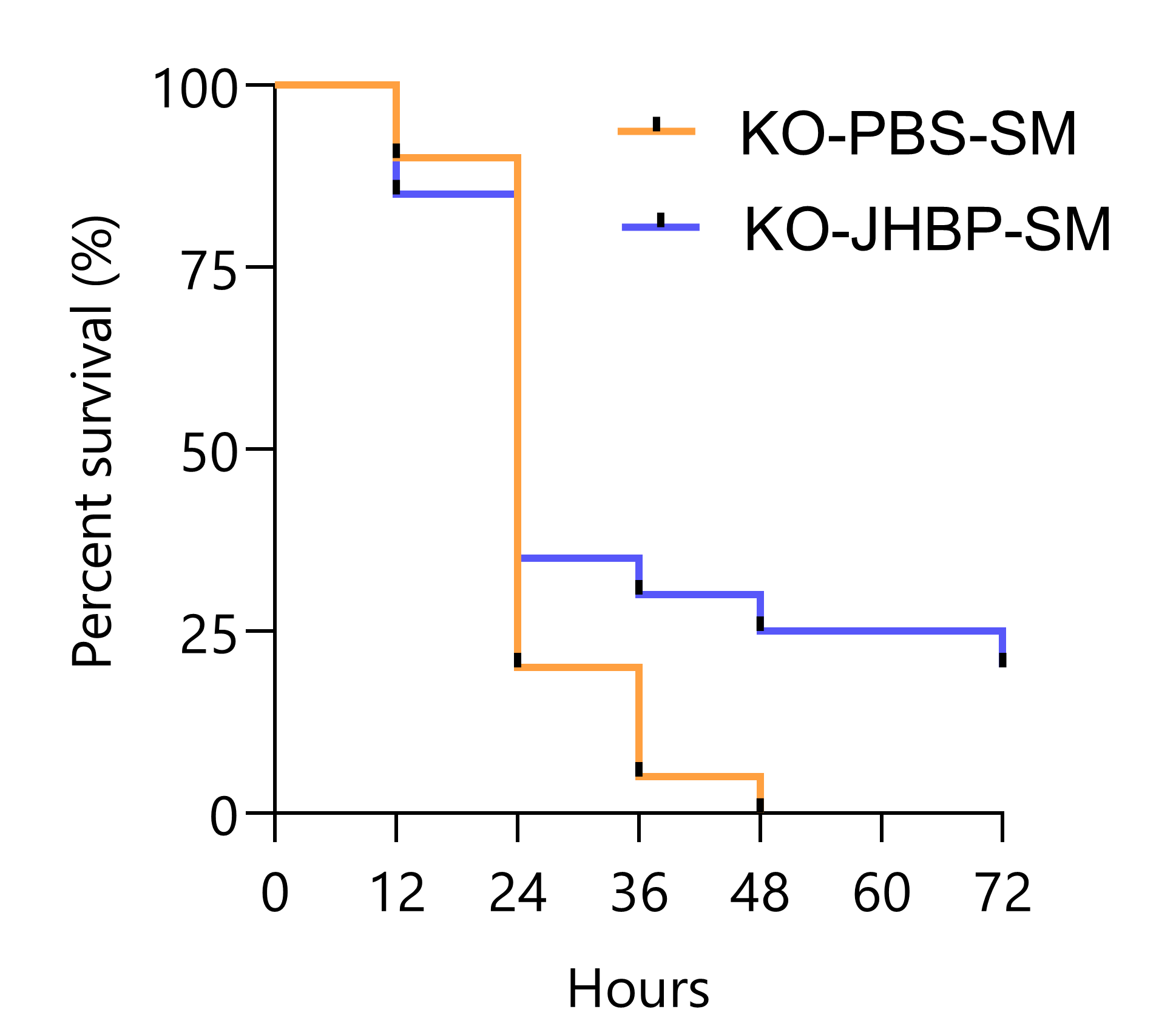

Supplement: S5 Fig — Kaplan-Meier survival curves for WT (n = 20) and KO (n = 17, two biological replicates) females infected by S. marcescens (OD600 = 0.05) two days after injection with 15 ng of mJHBP in 69 nL or 69 nL of PBS. The data were analyzed using the log-rank test as well as a fit to an accelerated time failure model with a Weibull distribution [15]. The results are described in the text. (TIF) [file ppat.1008288.s005.tif]

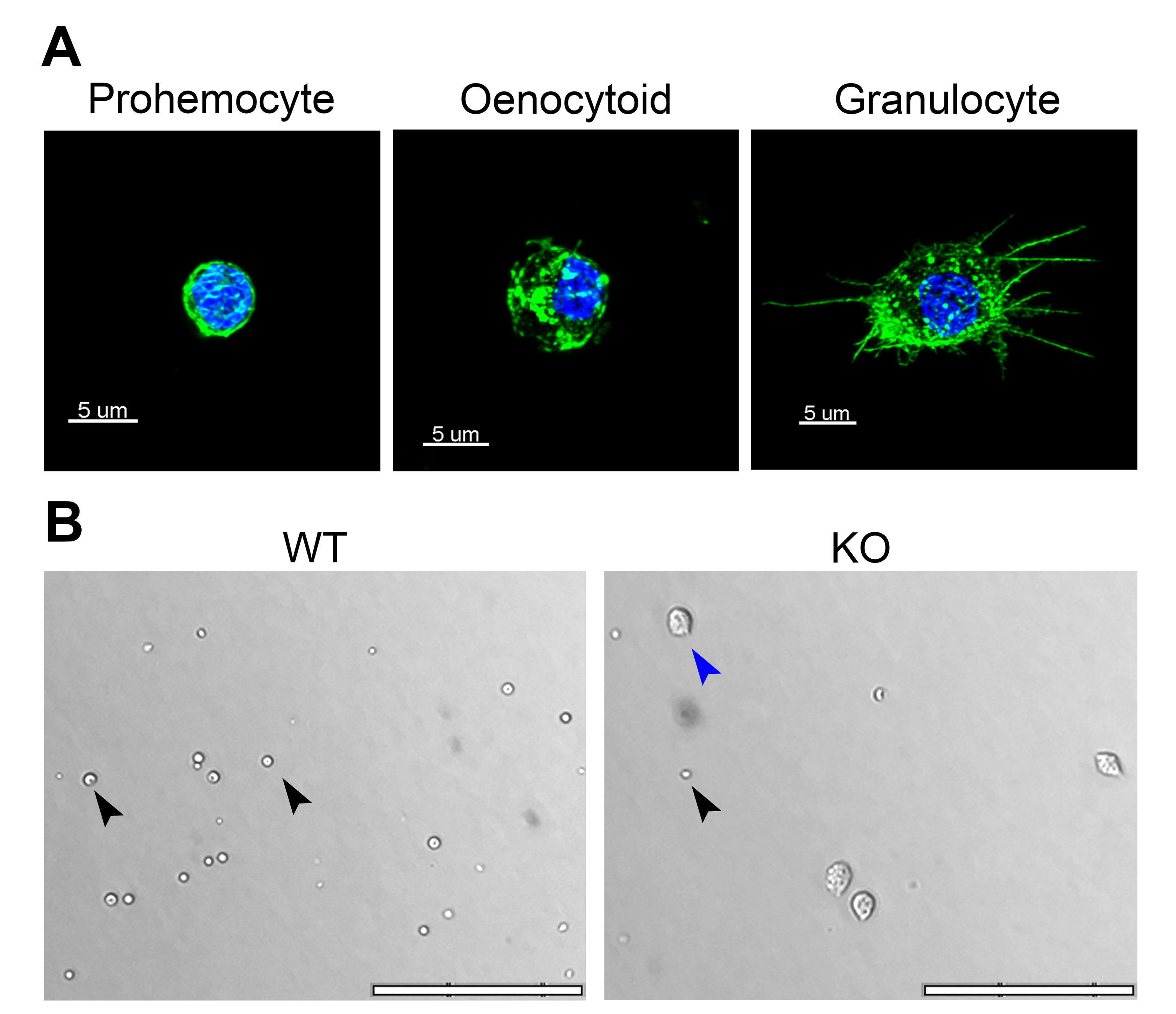

Supplement: S6 Fig — A. Confocal images of Ae. aegypti hemocytes stained with phalloidin (green) and Hoechst 33342 (nuclei, blue). Three distinct morphologies were identified: prohemocytes were small and showed no spreading, oenocytoids were circular in shape, larger and showed no spreading, while granulocytes were also large but showed spreading and contained visible inclusions. Prohemocytes have a higher nuclear to cytoplasmic ratio compared to oenocytoids. Maximum projection image. Scale bar = 5 μm. B. Low magnification bright field images of hemocytes in a hemocytometer collected from WT and KO mosquitoes by perfusion as described in the Materials and Methods. Black arrows indicate prohemocytes and blue arrows, granulocytes. Scale bar = 110 μm. (TIF) [file ppat.1008288.s006.tif]

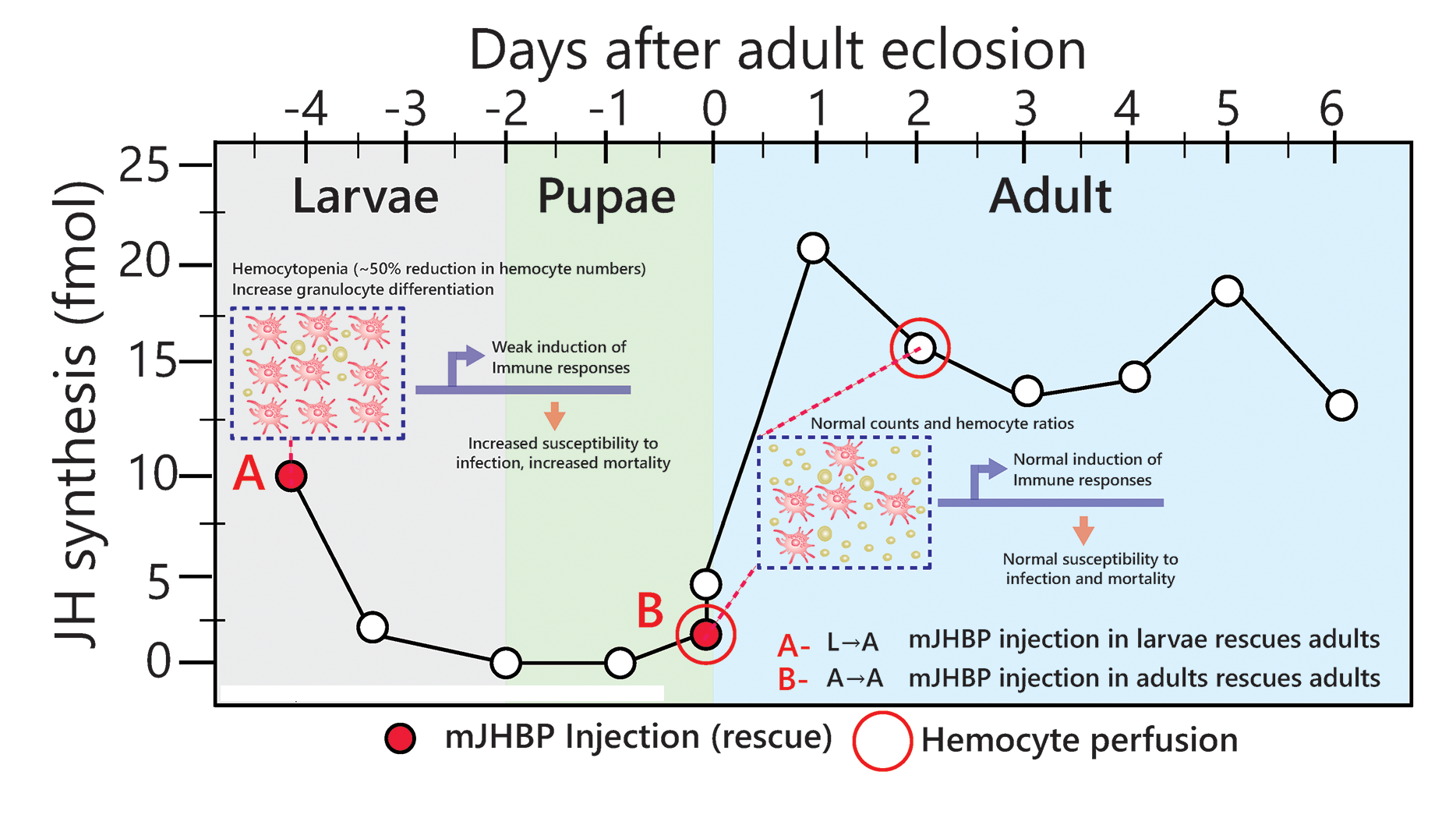

Supplement: S7 Fig — In the KO mutant, low hemocyte numbers and an altered hemocyte population structure lead to weak initial immune responses to bacterial infection and increased susceptibility to bacterial pathogens. JH levels are low in the pupal stage when mJHBP expression is initiated, but after the adult molt they increase dramatically and function in the previtellogenic development of the ovary. At this point mJHBP would be loaded with JHIII which may trigger stimulation of hematopoiesis and immunity. We know that injection of recombinant protein into the late larval stage or the adult stage (marked in diagram) rescues either normal hemocyte development or a normal antibacterial immune response, or both. Experiments with site-directed mutants suggest that JH binding may be essential for mJHBP function in these systems. (TIF) [file ppat.1008288.s007.tif]

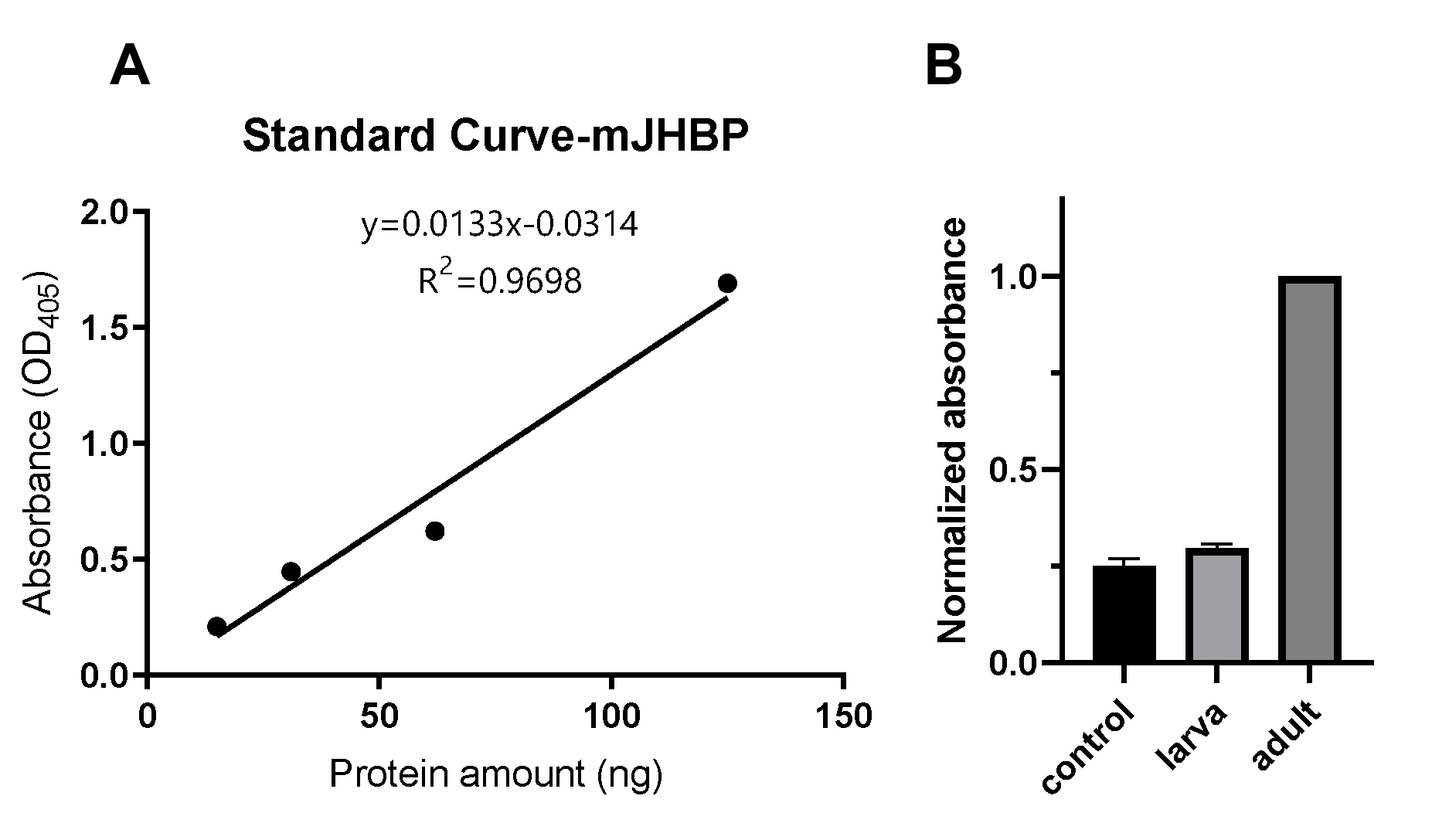

Supplement: S8 Fig — A. Standard curve used for the mJHBP quantifications by ELISA. Recombinant mJHBP was diluted to final concentrations of 125, 62, 31, and 15 ng and the OD405 of each concentration was measured as described in the Materials and Methods. B. Comparison of ELISA signal from control (BSA alone, 15 μg), larval homogenate (16 μg protein), adult homogenate (13 μg). The signal from larval homogenates was similar to the control. (TIF) [file ppat.1008288.s008.tif]
