# Supplementary material for: A mosquito juvenile hormone binding protein (mJHBP) regulates the activation of innate immune defenses and hemocyte development
Source: PLoS Pathog. 2020 Jan 21;16(1):e1008288. doi: 10.1371/journal.ppat.1008288 (PMC6994123; doi:10.1371/journal.ppat.1008288)
Supplement: S1 Table — (DOCX) [file ppat.1008288.s009.docx]

**S1 Table**. Primers used in this study

| **Primers** | **VectorBase #** | **5’ to 3’ Sequence** | **Use** |
| --- | --- | --- | --- |
| AaS7 F | AAEL013731 | GGGACAAATCGGCCAGGCTATC | qPCR reference |
| AaS7 R | AAEL013731 | TCGTGGACGCTTCTGCTTGTTG | qPCR reference |
| AaCecropin A F | AAEL029038 | ATTTCTCCTGATCGCCGTGGCTG | qPCR |
| AaCecropin A R | AAEL029038 | GAGCCTTCTCGGCGGCATTGAA | qPCR |
| AaDefensin A F | AAEL003841 | GCCACCTGTGATCTGTTGAGCGGA | qPCR |
| AaDefensin A R | AAEL003841 | GGAGTTGCAGTAGCCTCCCCGAT | qPCR |
| AaAttacin B F | AAEL003389 | CAACACATTGCTGTTCACTTTCGT | qPCR |
| AaAttacin B R | AAEL003389 | TTGGAAGTTGTTACCTGGAGTGAG | qPCR |
| AaGambicin F | AAEL004522 | TTGCCTTTACCTTGCCTACAGAGT | qPCR |
| AaGambicin R | AAEL004522 | GGCATAAACAAACACCAAAGCATC | qPCR |
| AaNOS F | AAEL009745 | TACGACTCGAGGGGCTAAAAG | qPCR |
| AaNOS R | AAEL009745 | CGCAGAATATCACACCCAACAA | qPCR |
| AaIMD F | AAEL010083 | TTCGGCAGATGATGGAGTGTGAGG | qPCR |
| AaIMD R | AAEL010083 | TGCGCGACCAGTCCAAAATGAAC | qPCR |
| AaCaspar F | AAEL027860 | CGCCGCATGACGTAGTTTTTAGG | qPCR |
| AaCaspar R | AAEL027860 | TTTCTGTTTACCCGGTTCCCATCC | qPCR |
| AaMyD88 F | AAEL007768 | AACGAGCATTGGAGCGGAT | qPCR |
| AaMyD88 R | AAEL007768 | TCAAAAGTAATACCGGCAAGAAGA | qPCR |
| AaCactus F | AAEL000709 | GGGCAGAACTTCAGCTCGGACAAT | qPCR |
| AaCactus R | AAEL000709 | CGCTGCTCGGGTTCTGCTGACT | qPCR |
| AaSTAT-B F | AAEL020559 | TCGAGGTGACCAATCTGCTGAA | qPCR |
| AaSTAT-B R | AAEL020559 | CTGCGTGGCCTGTGCTTGTG | qPCR |
| AaRel1A F | AAEL007696 | AATACGGCGAGTTCCAGCATAC | qPCR |
| AaRel1A R | AAEL007696 | TTCCGGAGAGTCATTTTTCAGT | qPCR |
| AaRel2 F | AAEL007624 | GAATCTGTATCGCCCGTGTCGTCA | qPCR |
| AaRel2 R | AAEL007624 | CGGGTAGGCGTTGGAGATGTTCA | qPCR |
| AaPGRP-S1 F | AAEL009474 | AGGCGGTGCTTCTTGTTGTGAT | qPCR |
| AaPGRP-S1 R | AAEL009474 | AGCTTTGCGTTGCCGTATGGTG | qPCR |
| AamJHBP F |  | NNNCATATGAGCGCTATCCCTTGTGAGGGTCAG | Site-directed mutagenesis |
| AamJHBP TAA R |  | NNNCTCGAGTTAAAAATAAAAGGCCGTCTTCTGGTTGCGATCG | Site-directed mutagenesis |
| AamJHBP 5’ YI R |  | CGCCGTTACCCAGTTTCGCATACGTGTTACCCGCGTCACGCACGTCGCCAT | Site-directed mutagenesis |
| AamJHBP Aae 3’ YI F |  | CCATCCTGCGTATGTTCCATGGCGACGTGCGTGACGCGGGTAACACGTAT | Site-directed mutagenesis |
| AamJHBP 165I 3’ F |  | GTCGGTGCGTTTCTCGCAGAAGTCCACGAACATCTGGCCTTTCTGTTTAACGCCG | Site-directed mutagenesis |
| AamJHBP 165I 5’ R |  | GTCGGTGCGTTTCTCGCAGAAGTCCACGAACATCTGGCCTTTCTGTTTAACGCCG | Site-directed mutagenesis |
